# De Novo Mutation Rate Variation and Its Determinants in *Chlamydomonas*

**DOI:** 10.1093/molbev/msab140

**Published:** 2021-05-05

**Authors:** Eugenio López-Cortegano, Rory J Craig, Jobran Chebib, Toby Samuels, Andrew D Morgan, Susanne A Kraemer, Katharina B Böndel, Rob W Ness, Nick Colegrave, Peter D Keightley

**Affiliations:** 1 Institute of Evolutionary Biology, School of Biological Sciences, University of Edinburgh, Edinburgh, United Kingdom; 2 Department of Biology, Concordia University, Montreal, QC, Canada; 3 Institute of Plant Breeding, Seed Science and Population Genetics, University of Hohenheim, Stuttgart, Germany; 4 Department of Biology, University of Toronto Mississauga, Mississauga, ON, Canada

**Keywords:** *Chlamydomonas incerta*, *Chlamydomonas reinhardtii*, comparative mutability, mutation accumulation, mutation rate, mutation spectrum

## Abstract

De novo mutations are central for evolution, since they provide the raw material for natural selection by regenerating genetic variation. However, studying de novo mutations is challenging and is generally restricted to model species, so we have a limited understanding of the evolution of the mutation rate and spectrum between closely related species. Here, we present a mutation accumulation (MA) experiment to study de novo mutation in the unicellular green alga *Chlamydomonas incerta* and perform comparative analyses with its closest known relative, *Chlamydomonas reinhardtii*. Using whole-genome sequencing data, we estimate that the median single nucleotide mutation (SNM) rate in *C. incerta* is *μ* = 7.6 × 10^−10^, and is highly variable between MA lines, ranging from *μ* = 0.35 × 10^−10^ to *μ* = 131.7 × 10^−10^. The SNM rate is strongly positively correlated with the mutation rate for insertions and deletions between lines (*r *>* *0.97). We infer that the genomic factors associated with variation in the mutation rate are similar to those in *C. reinhardtii*, allowing for cross-prediction between species. Among these genomic factors, sequence context and complexity are more important than GC content. With the exception of a remarkably high C→T bias, the SNM spectrum differs markedly between the two *Chlamydomonas* species. Our results suggest that similar genomic and biological characteristics may result in a similar mutation rate in the two species, whereas the SNM spectrum has more freedom to diverge.

## Introduction

Mutation plays a key role in evolution, since it generates genetic variation, providing the raw material for selection and adaptation. New mutational variance and heritability are important for determining the long-term response to selection (Walsh 2004; Mulder et al. 2019), and thus the evolutionary potential of populations. Standing genetic variation is also strongly influenced by variants continuously regenerated by mutation, so that heritability under mutation-drift equilibrium depends directly on the input of mutational variation (Lynch et al. 1999; Walsh and Lynch 2018). Since mutation also underlies genetic differentiation between lineages, it influences evolutionary divergence rates (Keightley 2012). Moreover, when new mutations have direct effects on phenotypes, particularly fitness and health, they also have a major impact in applied fields, such as conservation biology and medicine (Charlesworth 2018; Zhang and Vijg 2018). A better understanding of the rate of mutation and the distribution of mutational effects is one of the key goals in evolutionary biology (Eyre-Walker and Keightley 2007).

From an evolutionary perspective, the mutation rate itself can be regarded as a quantitative trait, which is modulated by natural selection and genetic drift (Lynch and Conery 2003; Lynch 2010). The drift-barrier hypothesis proposes that selection drives mutation rates to low values, minimizing the deleterious load, and that this process is more efficient in populations of higher effective size (*Ne*) (Lynch 2011; Sung, Ackerman, et al. 2012). However, although *Ne* is expected to play a central role in mutation rate evolution, there are likely to be many other genetic and biological factors that are involved in the evolution of mutation rates and its differentiation between species, such as the size of the functional genome (Sung, Ackerman, et al. 2012) and an organism’s life cycle (Sung, Tucker, et al. 2012; Long et al. 2015). The mutation rate is also affected by environmental conditions (Bjedov et al. 2003), and its evolution could in part be driven by factors including spatial and temporal heterogeneity (de Visser 2002). From a genomic point of view, the mutation rate has been observed to be highly heterogeneous along the genome, and many factors contribute to genomic variation in mutability, including nucleotide context, GC content, and DNA repetitiveness, among others (Mirkin 2008; Stamatoyannopoulos et al. 2009; Muzny et al. 2012; Aggarwala and Voight 2016; Sanjuán and Domingo-Calap 2016; Sassa et al. 2016; Frigola et al. 2017; Leffak et al. 2017; Kessler et al. 2020; McKinney et al. 2020). However, whether or not these correlates of mutation rate are causal, how they compare across species and the extent of their involvement in the evolution of the mutation rate remain open areas of study.

Because individual mutations are very rare, we have only recently been able to study large sets of mutations by combining whole-genome sequencing (WGS) and mutation accumulation (MA) experiments (Katju and Bergthorsson 2019). In an MA experiment, inbred or clonal lines are maintained with minimal *Ne* so that selection is ineffective and newly arising mutations can drift to fixation. This approach has been used in a variety of organisms (Keightley et al. 2009; Denver et al. 2012; Zhu et al. 2014; Flynn et al. 2017; Hamilton et al. 2017; Long et al. 2018; Krasovec et al. 2019; Weng et al. 2019; Kucukyildirim et al. 2020; Chebib et al. 2021), but has been generally limited to phylogenetically distant model organisms and rarely applied in closely related species to enable comparative investigation of mutation.

For example, in the distantly related yeast species *Saccharomyces cerevisiae* and *Schizosaccharomyces pombe*, Farlow et al. (2015) observed similar mutation rates, but different spectra of single nucleotide mutations (SNMs). Although the similarity of the mutation rates agreed with the drift-barrier expectation, these yeast species are so distantly related that their genomes share essentially no detectable synteny (Rhind et al. 2011), and a comparative analysis on their mutation properties provide little insight into the phylogenetic scale over which changes in the mutation spectrum evolves. Denver et al. (2012) addressed the importance of estimating mutational properties in more closely related species by studying several genotypes of two *Caenorhabditis* species that diverged approximately 100 Ma (Stein et al. 2003). In their MA experiment, neither the mutation rate nor SNM spectra differed significantly among species or genotypes, indicating that the mutation rate and spectrum may be evolutionarily stable in this clade. More recently, Terekhanova et al. (2017) analyzed sequence data from human and other primates and showed that estimates of the mutation rate in shared genomic windows (i.e., local mutation rates) are similar between closely related species (e.g., human and chimpanzee), but the correlation between mutation rate estimates decays with phylogenetic distance. Nonetheless, the scale over which the mutation rate and spectrum diverge remains unclear, and more information on the evolution of mutation processes in phylogenetically closely related species is needed.

Here, we present an MA experiment in *Chlamydomonas incerta*, the closest known relative of the green alga *Chlamydomonas reinhardtii*, which has emerged as a model for the study of rate and fitness effects of de novo mutation (Ness et al. 2012; Sung, Ackerman, et al. 2012; Morgan et al. 2014; Ness et al. 2015; Kraemer et al. 2017; Böndel et al. 2019). Although the taxonomic classification of *C. incerta*, which is also referred to as *C. globosa*, is subject to ongoing debate, it has long been recognized as a genetically and biologically distinct species from *C. reinhardtii* (Pröschold et al. 2005; Popescu et al. 2006; Nakada et al. 2010). The two species exhibit ∼34% divergence at 4-fold degenerate sites, likely diverged less than 100 Ma and have highly syntenic genomes with similar gene contents (Craig et al. 2021). A highly contiguous and well-annotated *C. incerta* genome assembly has recently been produced using Pacific Biosciences sequencing, enabling comparative genomics analyses between *C. reinhardtii* and its closest relative to be performed for the first time (Craig et al. 2021). Thus, a comparative study on the mutation rate, its spectrum, and the genomic factors related to mutability should lead to a better understanding of the mutation process and its evolution. Based on predictive statistical models, the nearly 6,000 mutations identified in *C. reinhardtii* by Ness et al. (2015) provided several insights into the genomic factors related to mutability. Here, we also study the *C. reinhardtii* data from Ness et al. (2015) together with nearly 2,000 new SNMs identified in *C. incerta* and investigate the evolution of mutational properties in the two species*.* Specifically, we address the following questions: 1) is the rate and base spectrum similar between the two species? 2) Is the extent of mutation rate variation between lines and across the genome similar in the two species? 3) Are predictors of mutation rate variation in the genome similar in the two species?

## Results

### Mutation Rates

A total of 27 MA lines of *C. incerta* were maintained for an average of 788 generations before performing whole-genome resequencing. The analysis of the re-sequencing data aligned to the *C. incerta* reference genome (129.2 Mb) gave an overall proportion of 72% high-quality sites (i.e., callable sites), where candidate mutations could be called. The fraction of callable sites (the callable rate) was consistent across MA lines (Kruskal–Wallis, KW test, χ^2^_26_ = 30.41, *P* = 0.25), but varied significantly between contigs (KW test, χ^2^_115_ = 2.6 × 10^6^, *P* < 2.2 × 10^−16^), and there was generally a higher callability in larger contigs (supplementary fig. S1*a–c*, Supplementary Material online) and the plastid (82%) and mitochondrial genomes (92%). The positive relationship between contig length and callability is likely to be a consequence of the highly repetitive content of many short contigs (Craig et al. 2021), which foil assembly and lead to low mapping quality (MQ) due to read misalignments (supplementary fig. S1*b*, Supplementary Material online). Although *C. incerta* has a higher mapped repeat content than *C. reinhardtii*, its callable rate is higher than that previously repeat content (∼28% vs. ∼22%) than the smaller *C. reinhardtii* genome (∼111 Mb), its callable rate is higher than that previously obtained in several strains of *C. reinhardtii* (Ness et al. 2015). This is presumably the result of the *C. incerta* MA lines being derived from the same strain as was used to produce the genome assembly, unlike in *C. reinhardtii* where field isolates exhibiting substantial genetic variation relative to the reference genome were studied. The callable rate was variable between different classes of genomic sites (supplementary fig. S1*d*, Supplementary Material online, KW test, χ^2^_4_ = 7.2 × 10^6^, *P* < 2.2 × 10^−16^) and was negatively correlated with the proportion of repetitive sequence (Pearson’s product-moment correlation, *t_3_* = −3.56, *r* = −0.90, *P* = 3.8 × 10^−2^).

Based on the callable portion of the *C. incerta* genome (∼84 Mb), a total of 2,609 de novo mutations were found, leading to an average mutation rate estimate per site per generation of *μ *≈ 15.10 × 10^−10^. There were 1,991 SNMs (*μ*_SNM_ = 11.56 × 10^−10^), 350 deletions (*μ*_DEL_ = 2.03 × 10^−10^), and 268 insertions (*μ*_INS_ = 1.56 × 10^−10^). These numbers are similar to those previously obtained in *C. reinhardtii*, that is, Ness et al. (2015) estimated *μ*  =  11.5 × 10^−10^ (*μ*_SNM_ = 9.63 × 10^−10^, *μ*_INS_ + *μ*_DEL_ = 1.90 × 10^−10^), with an average number of SNMs detected per line and generation similar to the number in the present experiment (∼9.72 × 10^−2^ in *C. incerta* vs. ∼7.15 × 10^−2^ in *C. reinhardtii*). The median *μ*_SNM_ = 7.62 × 10^−10^ in *C. incerta* was also similar to that of *C. reinhardtii* (*μ*_SNM_ = 5.27 × 10^−10^) and fell within the range observed for *C. reinhardtii* strains (Ness et al. 2015). In contrast, the average number of insertion and deletion variants (INDELs) per line and generation was higher in *C. incerta* (3.02 × 10^−2^ vs. 1.41 × 10^−2^), but this could be caused by differences in the callability of these variants (see below). No mutations were found in the mitochondrial genome, presumably because of its small size (∼17.6 kb), and only two SNMs were found in the plastid genome (*μ*_PLASTID_ = 5.4 × 10^−10^), resulting in an estimate of the mutation rate that is similar to that observed in *C. reinhardtii* (*μ*_PLASTID_ = 7.7 × 10^−10^). The plastid genome mutation rate is similar to the nuclear genome mutation rate in these species, a finding that contrasts with land plants (Smith and Keeling 2015; Ness et al. 2016). We restrict all further analyses to mutations found in the nuclear genome. Supplementary table S1, Supplementary Material online contains a list of all SNMs and INDELs found.

**Fig. 1.**
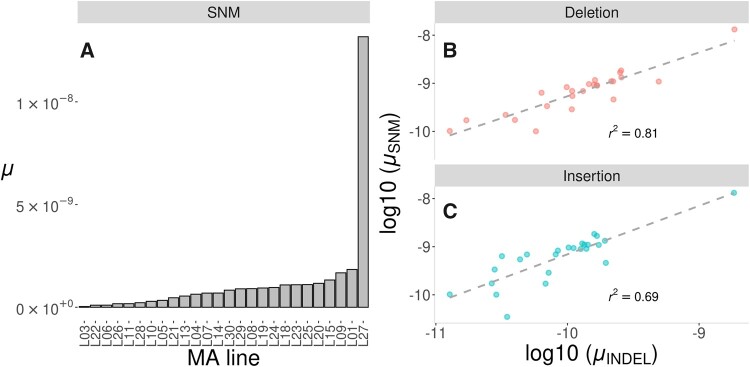
(A) Mutation rate (*μ*) estimates for SNMs in the *C. incerta* MA lines. Lines are sorted from lowest (left, *μ* = 0.04 × 10^−9^) to highest *μ* (right, *μ* = 13.2 × 10^−9^). (*B* and *C*) Correlation between *μ_S_*_NM_ and *μ*_INDEL_ (on log10 scale) for deletions (*B*, in red) and insertions (*C*, in blue). The dashed lines show linear regression lines for *μ*_SNM_ as a function of *μ*_INDEL_. Note the squared correlation coefficient is lower than that mentioned in the main text, because of the use here of a logarithmic scale.

Although the *C. incerta* MA lines were derived from a single ancestral strain, the mutation rate was highly variable among lines, ranging over more than two orders of magnitude, between *μ*  =  0.35 × 10^−10^ in line 3 with only 3 SNMs, to *μ*  =  131.7 × 10^−10^ in line 27 with 829 SNMs (fig. 1*A*). The distribution of the mutation rate among MA lines was highly leptokurtic (supplementary fig. S2*a*, Supplementary Material online), fitting better a lognormal distribution (meanlog = −3.01, sdlog = 1.15, Kolmogorov–Smirnov test, KS test, *D* = 0.14, *P* = 0.60) than any other distribution tested, including an exponential or gamma distribution. High variability among lines derived from the same genetic background was also observed in *C. reinhardtii* (Ness et al. 2015, see supplementary fig. S2*b*, Supplementary Material online), and the distribution of mutation rates in MA lines derived from *C. reinhardtii* strains CC-2344 and CC-2931 resembles that of *C. incerta* (KS test, *D* < 0.4, *P* > 0.07, supplementary fig. S2*b*, Supplementary Material online). After excluding hypermutant line 27, the variance in the number of mutations between lines of *C. incerta*, assuming an equal number of generations between MA lines, was still substantial (σ^2^_*μ*_ = 1070.03). This variation is approximately 22-fold higher than that expected from a Poisson distribution (λ  =  48.44, KS test, *D* = 0.38, *P* = 1.45 × 10^−3^), a distribution that is commonly assumed to represent the mutation processes (Charlesworth 2012). We also compared the observed distribution of mutation rates among MA lines with that obtained from computer simulations in which mutations arose independently, using the software SLiM (Haller and Messer 2019). The variance in the number of mutations among simulated lines was much closer to the Poisson expectation (median variance over replicates = 75.05, 95% CI = 39.87–128.04) than to the observed variance among the MA lines. High mutation rate variability between lines derived from the same ancestor genotype has been observed in other species (Dumont 2019; Ho et al. 2020) and supports the idea that substantial variability of the mutation rate may be common. Additional results and discussion on mutation rate variability and the hypermutant line 27 can be found in supplementary file S1, Supplementary Material online.

The mutation rate also varied significantly between contigs (KW test, χ^2^_115_ = 158.86, *P* = 4.24 × 10^−3^) and was generally higher and more variable in shorter contigs (Pearson’s product-moment correlation, *t_108_* = −2.59, *r* = −0.24, *P* = 0.01, supplementary fig. S3*a*, Supplementary Material online). Interestingly, the mutation rate also varied between gene-related features (KW test, χ^2^_4_ = 49.54 × 10^6^, *P* = 4.50 × 10^−10^). For example, the mutation rate was 40–50% higher in untranslated regions (UTRs) and intergenic regions than in coding regions, whereas intronic sequences had an intermediate rate (supplementary fig. S3*b*, Supplementary Material online). In principle, a lower number of mutations in coding sequences could be due to selection during the transfer of colonies from one plate to another. However, the estimate of *Ka/Ks* in coding sequences was 0.84 (Fisher’s exact test, *P* = 0.07), suggesting that little selection occurred, and that other genomic factors were therefore responsible for the variation in the mutation rate between genomic features. One possibility is that the DNA repair machinery is more efficient at preventing mutations in coding regions compared with intergenic sequences, as previously demonstrated in *Escherichia coli* and *Arabidopsis thaliana* MA lines that were mutant for mismatch repair proteins (Lee et al. 2012; Foster et al. 2015; Belfield et al. 2018). Higher mutation rates in UTRs were also observed in *C. reinhardtii*, although the intergenic mutation rate was not higher than that of coding sequence (supplementary fig. S4, Supplementary Material online). The two species have very similar amounts of annotated coding (39.5 Mb *C. incerta*, 37.7 Mb *C. reinhardtii*) and UTR (4.0, 3.9 Mb, respectively) sequence, and the ∼18 Mb greater assembly size of *C. incerta* is largely associated with a relative increase in repetitive intergenic sequence (Craig et al. 2021). This may suggest that genome organization plays a role in the observed difference in intergenic mutation rates between the species, although we cannot exclude alternative explanations (e.g., differences in callable rates or annotation quality). Nonetheless, the common pattern observed for coding regions and UTRs supports the existence of a bias toward low mutation rate at coding regions in Chlorophyta, since similar results were observed in other algal species (Krasovec et al. 2017).

The distribution of inter-mutation distance (IMD) differed significantly from the distribution expected if SNMs occurred at random genomic positions (KS test, *D* = 0.11, *P* < 2.2 × 10^−10^). This was due to an over-representation of mutation pairs separated by less than 10 bp (supplementary fig. S5*a*, Supplementary Material online). Among these mutations, 17 SNM pairs out of 27 (63%) occurred at adjacent sites. The most highly represented dinucleotide mutation was CC (35%), which always mutated to AA, TA, or TT. C→A mutations were nearly 2-fold more frequent when compared with other SNM types at dinuleotide mutation sites, after correcting for GC content (supplementary fig. S5*b*, Supplementary Material online). Differences from random expectation in the number of mutations in genomic segments of different lengths (1, 10, 100, or 500 kb) were nonsignificant (KS test, *D* < 0.08, *P* > 0.1). These results are also in broad agreement with previous findings in *C. reinhardtii* (Ness et al. 2015), indicating not only that mutation rates are similar between *C. incerta* and *C. reinhardtii*, but that mutations are similarly distributed across the genome.

### INDELs and Structural Variants

Three different software packages were used to detect short INDELs and structural variants: Freebayes (Garrison and Marth 2012), GATK (Van der Auwera et al. 2013), and Pindel (Ye et al. 2009). In contrast to SNMs, many of the INDELs and structural variants detected could not be confirmed by visualization in IGV (Thorvaldsdóttir et al. 2013), or the visualized variants did not exactly correspond with the calls in the VCF files. Therefore, to minimize the number of false positives, INDELs, and structural variants were manually curated following visualization by IGV. On this basis, approximately 56% of deletions and 10% of insertions detected by Pindel were rejected, whereas only 6% and 14% of the deletions and insertions called by GATK were excluded. Only 1% of deletions found by Freebayes were rejected. In general, most INDELs were found using GATK, followed by Pindel and Freebayes (supplementary fig. S6, Supplementary Material online). A false-positive rate in *C. reinhardtii* was estimated from our previous data (Ness et al. 2015) as the percentage of mutations originally called in a line but not verifiable in a visible inspection prior to genotyping them in recombinant lines (Böndel et al 2019). This rate suggested more false positives for INDELs (7.7%) than SNMs (2.3%), and it is expected to be of the same magnitude in *C. incerta*, given the similar approach used for sequencing, alignment, and calling.

After filtering, deletions were significantly more frequently retained than insertions (χ^2^_1_ = 10.84, *P* < 0.001), and the estimated mutation rates were *μ*_DEL_ = 2.03 × 10^−10^ for deletions and *μ*_INS_ = 1.56 × 10^−10^ for insertions. However, it should be noted that the short-read sequencing used here makes deletions of all sizes easier to detect than insertions. The INDEL mutation rate was higher in *C. incerta* than in *C. reinhardtii* (*μ*_DEL_ = 1.03 × 10^−10^, *μ*_INS_ = 0.87 × 10^−10^, Ness et al. 2015), even when variants only called by GATK were considered (*μ*_DEL_ = 1.58 × 10^−10^, *μ*_INS_ = 1.40 × 10^−10^). This was the only variant caller used to analyze the *C. reinhardtii* data.

The numbers of deletions and insertions were highly variable among MA lines, but were strongly positively correlated with the number of SNMs (Pearson’s product-moment correlation, *t_24_* > 18, *r*_SNM-DEL_ = 0.97, *r*_SNM-INS_ = 0.99, *P* < 1 × 10^−15^; fig. 1*B* and *C* and supplementary fig. S7*a*, Supplementary Material online), even after excluding the hypermutant line (*t_23_* > 5, *r*_SNM-DEL_ = 0.72, *r*_SNM-INS_ = 0.78, *P* < 1 × 10^−4^). This suggests that the mechanisms responsible for the occurrence of SNMs and INDELs are related. Deletions were generally larger than insertions (Wilcoxon rank-sum test, *W* = 58,003, *P* = 1.49 × 10^−8^, supplementary fig. S7*b*, Supplementary Material online), that is the median length was 1 bp for insertions and 2 bp for deletions, although larger deletions were also detected. There were 47 deletions longer than 100 bp (*μ*  =  2.15 × 10^−11^), and 8 of them were longer than 10 kb (*μ*  =  3.66 × 10^−12^). Short INDELs (<50 bp) were slightly shorter than in *C. reinhardtii*, that is mean lengths were 3.6 bp for deletions and 2.3 bp for insertions (vs. 7.9 bp for deletions and for insertions 5.9 bp in *C. reinhardtii*). In addition to INDELs, five large inversions were found by Pindel (*μ*_INV_ = 2.2 × 10^−12^), with sizes ranging from ∼700 to ∼3,700 bp. No insertions or tandem repeat variants larger than 50 bp were found. As mentioned above, INDEL detection was more challenging that detection of SNM variants, so only SNMs will be considered for further analyses, unless otherwise stated.

### Factors Influencing Mutability

**Fig. 2.**
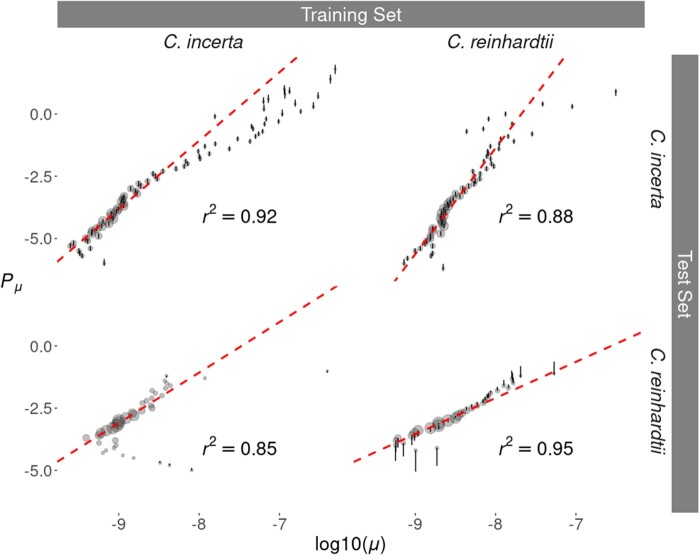
Correspondence of predicted and observed mutability within and across the *Chlamydomonas incerta* and *Chlamydomonas reinhardtii* genomes. Predicted mutability (*P_μ_*) was obtained for the *C. incerta* and *C. reinhardtii* validation data (top and bottom plots, respectively) using models trained with either *C. incerta* or *C. reinhardtii* data (left and right plots, respectively). Genomic regions were binned in groups with the same *P_μ_* value (rounded to 1 decimal place) and its value was compared with the observed mutation rate (*μ*) of the same sites. Vertical bars show 95% confidence intervals of *P_μ_* values obtained over ten replicates using different training data sets. Red dashed lines show the linear fit of the predictions, weighted by the number of sites in each bin (larger points indicate more observations). All fits were significant (*P* < 10^−3^), with coefficients of determination (*r^2^*) shown in the figure.

**Fig. 3.**
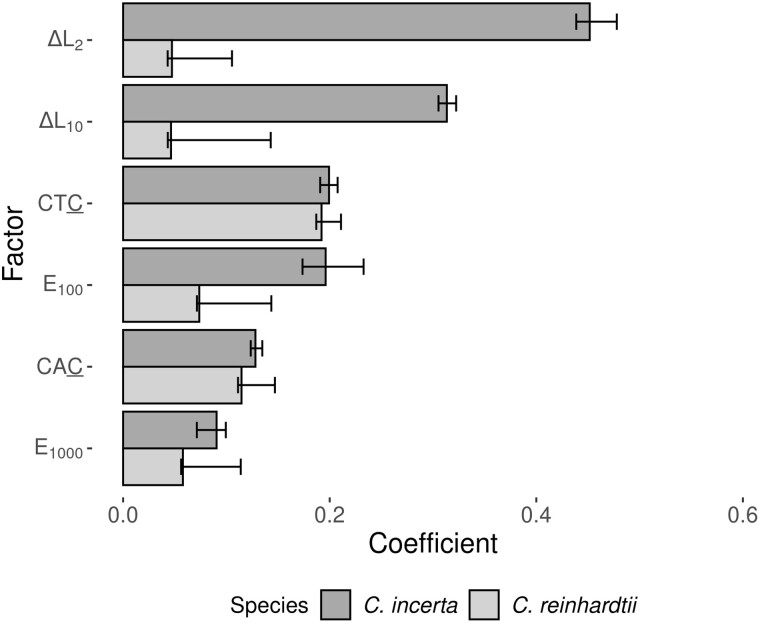
Regression coefficient estimates obtained from the predictive model of genomic mutability. Median estimates and 95% confidence intervals are shown based on ten training set replicates of *C. incerta* (dark bars) and *C. reinhardtii* (light bars). Only factors that were common between the ten most important ones estimated in *C. incerta* and *C. reinhardtii* are shown. The factors shown in the figure are: Variation in sequence repetitiveness (Δ*L*_2_ and Δ*L*_10_, measured in windows of 2 and 10 bp, respectively, extending downstream and upstream), nucleotide repetitiveness (*E*_100_ and *E*_1000_, measured in windows of 100 and 1,000 bp, respectively, extending downstream and upstream), and trinucleotides CTC and CAC (where the underlined C is the reference site containing the mutation). Factors are sorted by their median effect sizes estimated in *C. incerta*. All predictors were standardized prior to regression, so their effect sizes are comparable.

To examine the genomic factors associated with mutability, we employed a regularized logistic regression model to predict mutated sites from their genomic properties. The use of a regularized regression algorithm reduces the effect of correlation between the multiple predictor variables fitted in the model. For both *C. incerta* and *C. reinhardtii*, a training set composed of all identified SNMs along with a random sample of 10^5^ nonmutated callable sites was used for fitting the model. To test how well the model predicted mutability, a test set containing all SNMs and a larger set of 10^6^ randomly selected callable sites was used. A cross-validation scheme between species was implemented by running the model fitted to one species in order to predict mutability in the other. The predictive mutability model always returned statistically significant results, including for the crossed-predictions (*P* < 1 × 10^−3^). There was also a strong linear relationship between the predicted and the observed mutability, which was higher within species than between species (fig. 2). The correlation between the regression coefficient estimates in the two species was significantly positive (Pearson’s product-moment correlation, *t_141_* = 5.77, *r *=* *0.44, *P* = 4.86 × 10^−8^), and six out of the ten most important genomic factors associated with mutability were shared by *C. incerta* and *C. reinhardtii* (fig. 3). Supplementary figure S8, Supplementary Material online shows the ten most important predictors for *C. incerta* and *C. reinhardtii*, and a complete list including all raw regression coefficient estimates is given in supplementary table S2, Supplementary Material online. These results indicate that the mechanisms associated with mutation in the two species are highly related.

**Fig. 4.**
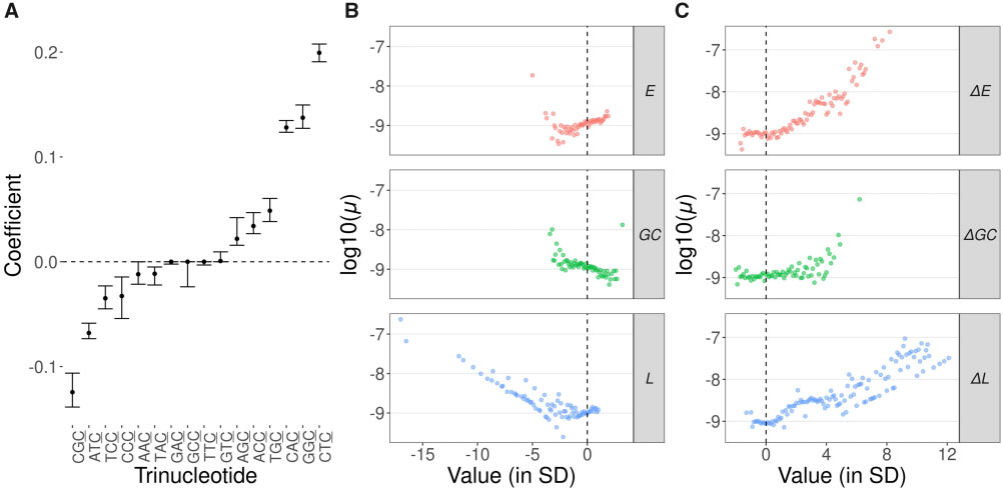
Relationships between sequence context, base composition, sequence complexity, and mutability in *C. incerta*. (*A*) Regression coefficient estimates for the 16 possible dinucleotides upstream of reference C sites. (*B*) Relationship between scaled mean nucleotide repetitiveness (*E*), GC content (*GC*), and sequence repetitiveness (*L*), measured in genomic windows of 2 Kb. (*C*) Relationship between the scaled variation in nucleotide repetitiveness (*ΔE*), variation in GC content (*ΔGC*), and variation in sequence repetitiveness (*ΔL*), measured in a genomic windows of 20 bp. Note that standard deviations are used as the unit of measurement for the genomic parameters in plots (*B*) and (*C*). Only genomic sections where *μ *> 0 are shown in (*B*) and (*C*).

**Fig. 5.**
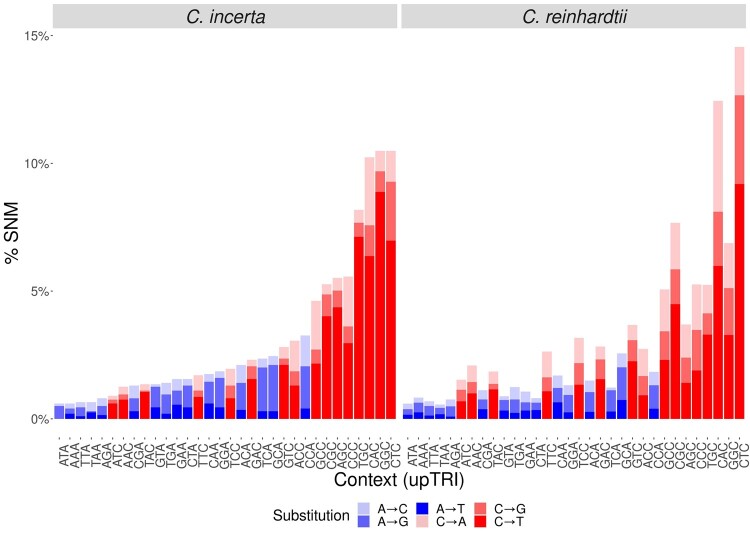
Percentage of different types of SNM by upstream genomic context, defined as 2 bp upstream from a reference A or C site. Bars are colored by nucleotide composition at the reference site (A/T: blue, C/G: red). For each bar the different types of SNM are further colored in light (A→C and C→A), intermediate (A→G and C→G) or dark (A→T and C→T) colors. Sequence context is sorted on the *x*-axis by mutation frequency in *C. incerta*.

Remarkably, the trinucleotides CTC and CAC (where the underlined C represents the mutated site) had a similar effect on mutability in the two species. The CTC trinucleotide was the single most important factor in *C. reinhardtii*, and its high mutability has been previously reported in this species (Ness et al. 2015), as well as in other phylogenetic groups, including fungi (Zhu et al. 2014) and animals (Alexandrov et al. 2013). This finding highlights the importance of sequence context variation for variation in mutability (Ling et al. 2020), which is supported by the large amount of variation in the effect of different triplet sequences on mutability (supplementary table S2, Supplementary Material online). For example, regarding the upstream context of C nucleotides, there were regression coefficients both positively and negatively associated with mutability in *C. incerta* (fig. 4*A*). In *C. reinhardtii*, most coefficient estimates related to sequence context were close to zero (supplementary fig. S9*a*, Supplementary Material online), probably due to differences in the penalty term of the regularized regression model in the two species. However, the observed proportion of SNMs in different sequence contexts (fig. 5) was highly variable in the two species, and strongly correlated between them (Pearson’s product-moment correlation, *t_93_* = 13.14, *r *=* *0.81, *P* < 10^−15^).

GC content is the variable most commonly used to reduce local genomic information into a single value, since it is typically correlated with important biological features, including the mutation rate (Krasovec et al. 2017). Here, we explored the role of two additional parameters in relation to mutation, the local Shannon Entropy (*E*) and sequence linguistic complexity (*L*), both of which provide insight into the amount of noncompressible information contained in DNA sequences (Schneider 2000; Vinga 2014). In simple terms, *E* measures nucleotide repetitiveness (it is maximal when all nucleotides have equal frequencies, *p *=* *0.25, and minimal when *p *=* *1 for one nucleotide), whereas *L* measures sequence repetitiveness (it is maximal in regions where all possible different sequences of a given length or range of lengths are represented). We also quantified GC content and variation in GC, *E* and *L* within genomic windows (named ΔGC, Δ*E*, and Δ*L*, respectively). Thus, although GC measures the mean GC content within a genomic window, ΔGC measures the extent of GC variation within that window. Most genomic regions are characterized by having high *L* and low Δ*L*, Δ*E*, and ΔGC. Conversely, mutated sites were usually located in regions with low *L*, high Δ*L*, Δ*E*, and ΔGC, or in regions with unusually high or low values of *E* or GC (fig. 4*B* and supplementary fig. S9*b*, Supplementary Material online). This agrees with previous results suggesting that deviations in GC content from the genomic equilibrium lead to increased mutability (Krasovec et al. 2019). Interestingly, predictors related to nucleotide and sequence complexity showed a stronger association with mutability than GC content in both species (fig. 3 and supplementary table S2, Supplementary Material online). For example, Δ*L* in small windows (≤20 bp) showed the strongest association with mutability in *C. incerta*, and *E* and *L* were generally more informative about mutability than measures based on GC in both species (supplementary fig. S8 and table S2, Supplementary Material online). Exceptionally, in *C. reinhardtii* GC content at a mutated site showed a strong association with mutability, probably related to the hypermutability of CTC and CAC trinucleotides, and to the higher probability of mutation at G:C sites than at A:T sites in this species (see below).

In view of previous results on the importance of nucleotide and sequence complexity measures for predicting mutability, we also estimated the correlation between the mutation rate and distance from repetitive sequence regions annotated as low complexity regions and microsatellites. However, correlations were nonsignificant for both low complexity regions (Pearson’s product-moment correlation, *t_361177_* = −0.34, *r* = −5.68 × 10^−4^, *P* = 0.73) and microsatellites (*t_20111_* = 0.30, *r *=* *2.14 × 10^−3^, *P* = 0.76). It should be noted that our annotation of repetitive sequences did not include large satellite DNA, because these regions are not expected to be callable.

**Fig. 6.**
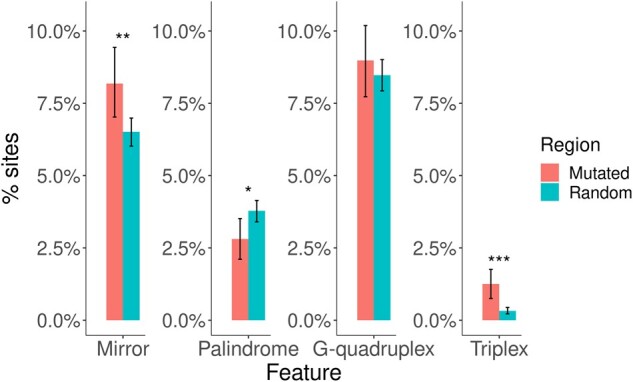
Percentage of genomic sites containing at least one of the following features: mirror, palindrome, G-quadruplex, and triplex in windows of 21 bp. Regions are grouped into those containing known mutated sites, containing a total of 1,991 SNMs (in red), and 10^4^ randomly sampled genomic locations (in blue). Significance is calculated using the Kruskal–Wallis test (*P < 0.05, **P < 0.01, ***P < 0.001). Confidence intervals (95%) are based on 1,000 bootstrap samples.

To further explore repetitive patterns that could help to explain the higher mutability of genomic regions of low linguistic complexity, we expanded our analysis to include alternate DNA conformations, which are usually characterized by repetitive sequences. This analysis was done using NeSSie (Berselli et al. 2018) and QPARSE (Berselli et al. 2020) to detect the absence/presence of potential motifs for DNA triplexes, G-quadruplexes, mirrors, and palindromes. When considering small genomic windows of 10 bp extending either side of a mutated or a randomly sampled site, the proportion of genomic regions containing potential DNA triplex motifs was approximately 4-fold higher in regions containing a mutated site than other genomic regions (KW test, χ^2^_1_ = 30.76, *P* = 2.92 × 10^−8^, fig. 6). Sequence mirrors, potentially associated with DNA hairpins (Gajarský et al. 2017), were also more frequently found near mutated sites (KW test, χ^2^_1_ = 7.95, *P* = 4.8 × 10^−3^), whereas palindromes were slightly under-represented (KW test, χ^2^_1_ = 4.51, *P* = 0.03). When analyzing genomic windows longer than 20 bp (0.2 and 2 Kb), no significant enrichment was found for any of the DNA sequence motifs considered (supplementary fig. S10, Supplementary Material online). Thus, it is possible that mutability is influenced by close proximity to DNA motifs that lead to alternate conformations such as DNA triplexes or hairpins. The bioinformatic analysis presented here, however, assumed that these motifs are truly indicative of the presence of DNA secondary structure in *C. incerta*, but there is no experimental confirmation, and therefore results should be interpreted with caution. More studies addressing sequence complexity and DNA secondary structure are needed, particularly in the context of de novo mutation.

### SNM Spectrum

**Fig. 7.**
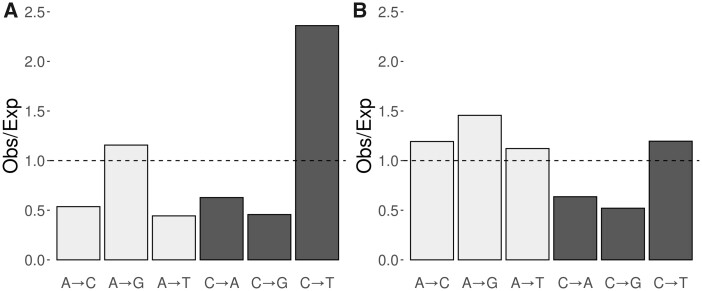
Spectrum of SNMs. (*A*) SNM spectrum for *C. incerta*. The height of the bars represents the deviation of the observed value from the expectation for all SNMs being equally frequent, after correcting for GC content (mean GC = 66%). (*B*) SNM spectrum for *C. incerta* relative to *C. reinhardtii*. The height of the bars is calculated using the observed/expected deviation calculated in (*A*) for *C. incerta*, divided by the same measurement estimated for *C. reinhardtii*, using data from Ness et al. (2015). Light bars refer to mutations at A:T sites, whereas dark bars refer to C:G sites.

The SNM spectrum departed from the expectation of equally frequent SNM types, after correction for the genomic mean GC content of 66% (χ^2^_5_ = 1,124.9, *P* < 2.2 × 10^−16^). Specifically, transition point mutations were much more frequent than transversions, and C→T transitions were 2.35 times more frequent than the random expectation, and twice as frequent as A→G transitions (fig. 7*A*). Overall, C→T mutations represented nearly 52% of all SNMs. A similar pattern was observed by Ness et al. (2015) in *C. reinhardtii.* However, whereas transitions were the most frequent mutation type in *C. incerta*, C sites had a higher mutability in *C. reinhardtii* (i.e., C→A transversions were more frequent than A→G transitions), mainly due to the hypermutability of the trinucleotide CTC. Consequently, the mutation spectrum of the two species is qualitatively different (fig. 7*B*).

As previously done for mutability, a regularized predictive model was run to determine the genomic factors that explain the occurrence of different types of SNMs. Thus, the data set only included observations at SNM sites, and the response variable was first defined as the SNM type, a multinomial variable with six levels, one per type of SNM (A→C, A→G, A→T, C→A, C→G, and C→T). Models tested included regularized multinomial regression and classification machine learning algorithms, such as neural networks and random forests (details on the tuning of the different models used are given in Materials and Methods). A random sample of 75% SNMs was used for training and the remaining 25% for testing, so that accuracy could be measured as the proportion of SNM types correctly classified in the test set, relative to the total number of observations. A cross-validation scheme between species was also included. For both species, however, all classification models considered produced low (<33%) and nonsignificant accuracy values. Alternatively, when the response variable was set as a binomial variable indicating whether or not an SNM was of type C→T, accuracy increased to values close to 65% both in *C. incerta* (exact binomial test, EB test, *P* < 1.6 × 10^−3^) and in *C. reinhardtii* (EB test, *P* = 0.02). Accuracy was always nonsignificant for predictions across species, and the most important predictors determining the occurrence of C→T mutations were different for each species (supplementary fig. S11 and table S3, Supplementary Material online). This indicates that the C→T bias is either associated with different factors in each species, the relative importance of the factors analyzed differ between the two species, or the most important explanatory variables regarding C→T prediction were not included in our model.

## Discussion

We have conducted a comparative study of mutability in *Chlamydomonas* green algae using WGS data from an MA experiment in *C. incerta* and from an experiment previously carried out in *C. reinhardtii* (Ness et al. 2015). Given their relatively large genomes (111–129 Mb) and their short generation intervals, these unicellular species represent excellent models for investigating the nature of de novo mutations, since large numbers of mutations can be accumulated in a short time (∼0.097 SNMs per line per generation), allowing the factors associated with the properties of new mutations to be investigated. Our analyses revealed that the mutation rate, the spatial distribution of mutations across the genome, and the genomic factors associated with mutability were similar between the two species, whereas the SNM spectra differed and were nonpredictable across species. The mutation rate was also found to be highly variable between lines derived from the same ancestral strain in both species.

It is perhaps unsurprising that the median SNM rate estimated in *C. incerta* (7.6 × 10^−10^) is similar to that observed across strains of *C. reinhardtii* (5.3 × 10^−10^, Ness et al. 2015), since they are closely related and have similar genomic architectures and total lengths of coding sequence. Earlier estimates of the mutation rate in *C. reinhardtii* were substantially smaller, that is 2.1 × 10^−10^ (Ness et al. 2012) and 0.7 × 10^−10^ (Sung, Ackerman, et al. 2012), but this difference could either be due to methodological differences in mutation detection or to between-strain variation in the mutation rate. Krasovec et al. (2017, 2018) estimated the mutation rate in five more distantly related green algal species that have much smaller genomes (in the range 12–21 Mb) and more variable GC contents (46–64%) than *Chlamydomonas*. In these species, estimates were nonetheless of the same order of magnitude as *C. incerta* and *C. reinhardtii* (3.02 × 10^−10^ ≤ *μ*SNM ≤ 9.19 × 10^−10^). Unfortunately, there are only two known isolates of *C. incerta* and no genetic diversity data are available for the species, so it is not currently possible to estimate *Ne* for the species, which can be used to test the “drift barrier” hypothesis (Sung, Ackerman, et al. 2012; Lynch et al. 2016).

Not only was the mutation rate similar between the two *Chlamydomonas* species, but also the genomic factors associated with mutability. There was a high correlation between the genomic predictors of mutability in the two species and an accurate cross-prediction of mutability between the species, suggesting that the modeled genomic factors were associated with the mutation rate in the species’ common ancestor. We found that sequence context and complexity were the most important factors associated with mutability in *Chlamydomonas*. The mutation rate varied substantially among different trinucleotide sequence contexts (fig. 5) and was especially high in the CTC and CAC contexts. Variation in sequence editing proteins with context-dependent activity, such as the APOBEC family of cytidine deaminases (Sanjuán and Domingo-Calap 2016), could explain the strong association of particular sequence contexts with mutability. For example, APOBEC3 is known to introduce C→T mutations and shows affinity for TC and CC substrates (Refsland and Harris 2013; Roberts et al. 2013). However, the true nature of the hypermutability of CTC and CAC contexts in *Chlamydomonas* remains unknown. Regarding measures of nucleotide and sequence complexity, these factors contributed more to the prediction of mutability than measures based on GC content alone (fig. 4 and supplementary fig. S9 and table S2, Supplementary Material online). The strong association between complexity-related measures and mutability might arise from the higher level of sequence information they provide compared with GC content. For example, nucleotide complexity (i.e., sequence entropy) is more sensitive to differences in the arrangement of nucleotides than GC content, and variation in complexity within genome sequences can be used to predict the presence of repetitive sequences (Thanos et al. 2018). Linguistic sequence complexity might be associated with nonstandard DNA conformations, which usually involve some degree of repetitiveness (Wells 1988; Bacolla and Wells 2004). High mutagenicity for secondary structure-forming sequences might then explain the association between low complexity sequences and mutability, for example via double-strand breaks (DSBs) or interference with the DNA repair machinery. This association has been described for Z-DNA, which is associated with DSBs and mutation in humans and yeast (McKinney et al. 2020), and whose presence seems to be associated with alternate purine-pyrimidine repeats. Although it is clear that higher-order patterns within sequences strongly influence stability and mutation rates, the causal mechanisms remain to be determined.

In spite of the close phylogenetic relationship between *C. reinhardtii* and *C. incerta*, and the aforementioned similarities of their mutation rates and associated genomic factors, the SNM spectra of the two species showed substantial differences. Only a high C→T bias was in common between the two SNM spectra, but this bias is nearly universal, since it has been found both in prokaryote and eukaryote organisms (Hershberg and Petrov 2010; Ossowski et al. 2010; Farlow et al. 2015; Krasovec et al. 2019). More generally, transitions are the most common SNM type in *C. incerta*, whereas in *C. reinhardtii* SNMs at C sites represent the most common type of SNMs. Although differences in the SNM spectra may evolve as a consequence of environmental change (Liu and Zhang 2019), we do not expect this to be the case here, since *C. incerta* and *C. reinhardtii* experiments were performed at the same time, under the same environmental conditions, and we have no evidence on these conditions being more stressful for one species than the other. In an evolutionary context, similar mutation rates, but different SNM spectra, have been observed in other taxa, such as in the yeast species *S. cerevisiae* and *S. pombe* (Farlow et al. 2015), and in diverse green algal species (Krasovec et al. 2017). However, previous studies have involved species that are phylogenetically more distantly related than *C. incerta* is to *C. reinhardtii*. Thus, our results highlight that the SNM spectrum may be subject to evolutionary divergence between closely related species, possibly contributing to the early differentiation of genomic and biological characteristics. Evolution of DNA repair machinery is likely to be related to the evolution of the SNM spectrum, as suggested by evidence from experiments in bacteria. For example, Dillon et al. (2017) showed that the SNM spectra in wild-type *Vibrio cholerae* and *Vibrio fischeri* strains are substantially different, but they converge in strains mutant for the DNA mismatch repair gene *mutS*. In *E. col*i, C→T mutations are the most common SNM, but in *mutL* mutants A→G mutations are the most abundant (Lee et al. 2012). Thus, it is possible that the differences between the *C. incerta* and *C. reinhardtii* SNM spectra have evolved as a consequence of a small number of substitutions in DNA repair genes, such as *mutS* homologs. Orthologs of known DNA repair genes have experienced nonsynonymous substitutions between the species (up to a rate of 2.4%, supplementary table S4, Supplementary Material online), and it is possible that some of these amino acid changes may contribute to SNM divergence in *Chlamydomonas*. However, confirming the involvement of DNA repair machinery divergence in shaping the SNM spectra would require experimental validation, making use of strains mutant for the DNA repair machinery.

The mutation rate and its spectrum are variable in nature and probably respond to the same evolutionary forces, including genetic drift and selection, as regular quantitative traits (Lynch 2010). In particular, the evolution of mutational properties is likely to be driven by the evolution of the DNA repair machinery. Understanding the genetic architecture of the mutation rate and the causes of its variation both at the population and the genomic level is a fundamental problem in evolutionary biology. Here, we have studied the determinants of the mutation rate in *Chlamydomonas*, and characterize differences in the SNM spectra of *C. incerta* and *C. reinhardtii*. The two species’ genomes are highly syntenic, but show an average synonymous divergence of ∼34% (Craig et al. 2021), which means they are on a similar scale of divergence as humans and rodents (Lindblad-Toh et al. 2011). We show that the mutation rate and its associated genomic factors have been maintained in *Chlamydomonas*, whereas the SNM spectra have substantially diverged, likely contributing to the appearance of genomic differences between species. Thus, our results contribute to the understanding of the evolution of mutational properties in closely related species. More work is needed, however, focusing on the evolution of mutational properties in the context of the evolution of the DNA repair machinery. Future research shall also benefit from using additional sources of de novo mutations, such as structural mutations, using recent long-read technology.

## Materials and Methods

### MA Experiment and WGS

MA lines were initiated from the *C. incerta* strain SAG 7.73, which was obtained from the SAG culture center (Germany). During the MA experiment, cell suspensions were spread out on Bold’s agar plates, and lines were bottlenecked regularly by picking single colonies at random and transferring them from one plate to another at intervals of 3–5 days for an average of 74 transfers. The effective population size was therefore expected to be low, reducing the effectiveness of natural selection. In order to calculate the number of generations that occurred during the MA experiment, the generation times of colonies growing over 3-, 4-, and 5-day periods were determined for two replicates of 14 of the MA line endpoints. Since the generation time is expected to increase as mutations accumulate, this procedure is likely to underestimate the generation time over the whole course of MA. However, the mean generation time of the MA lines was close to that measured for five replicates of the ancestor (Wilcoxon rank-sum test, *W* = 1,119, *P* = 0.71, supplementary fig. S12, Supplementary Material online), and therefore we expect this bias to be small. Liquid cultures were plated and colonies were allowed to grow for 3, 4, or 5 days. The total number of colonies (*N0*) on each plate was counted, and then the plates were flooded with medium in order to suspend the cells. Cell suspensions were diluted and replated, incubated for 5 days, and newly growing colonies counted. Then, the total colony forming units (*Nt*) in the undiluted cell suspensions were calculated. The number of generations (*t*) was computed as *t = (log Nt—log N0)/log 2*. Growth rate per day for each transfer period was then used to compute the total number of generations over the course of the entire MA experiment. For MA lines that did not have direct measurement of growth rate, the average rate of the other MA lines was used. Additional details on the maintenance of the MA lines can be found in Morgan et al. (2014).

A total of 27 lines were generated from the MA experiment. DNA was extracted from frozen cells using the phenol-chloroform protocol as described in Ness et al. (2012). Sequencing followed Ness et al. (2015). Briefly, whole-genome resequencing was performed at ∼25X coverage of 100 bp paired-end reads on the Illumina HiSeq 2500 platform by BGI (China), using modified PCR conditions (Aird et al. 2011) to accommodate the high GC content (66%) of the *C. incerta* genome.

### Alignment and Variant Calling

We used the ∼129 Mb *C. incerta* reference genome of the ancestral strain (SAG 7.73), which is a highly contiguous (contig-level N50 ∼ 1.6 Mb) assembly based on Pacific Biosciences sequencing (Craig et al. 2021). Plastid and mitochondrial genomes were included in the alignment. The short reads were aligned to the reference genome using BWA-MEM v.0.7.17 (Li and Durbin 2009). The resultant BAM files were sorted with SAMtools v.1.9 (Li et al. 2009; Li 2011) and further processed using Picard tools v.2.21.1 (Broad Institute 2019). The tool MarkDuplicates was used to tag duplicate reads, and AddOrReplaceReadGroups was used to update the files’ metadata. After processing, the average coverage measured with SAMtools was ∼21X.

To directly compare the mutations inferred in *C. incerta* with those from *C. reinhardtii*, contigs with no evidence of synteny between the two species were excluded. These represented approximately 9% of the *C. incerta* reference genome (Craig et al. 2021). Variant calling was first done using GATK v4.1.4.0 (McKenna et al. 2010; Van der Auwera et al. 2013), but variants found by Freebayes 1.3.2 (Garrison and Marth 2012) were also included. In GATK, the HaplotypeCaller command was used to generate a genome variant call format (GVCF) file for each line separately using the optional parameter for a haploid genome (-ploidy 1), and every genomic site was called, including nonvariant ones (-ERC BP_RESOLUTION). GVCF files were merged using GATK CombineGVCFs. The combined variant call format (VCF) file was indexed with IndexFeatureFile, and variants were called with GenotypeGVCFs (-ploidy, -all-sites). Freebayes was run with ploidy set to 1 (-p 1), and invariant sites (–report-monomorphic) were included in the output. In addition to GATK and Freebayes, Pindel v0.2.5b9 (Ye et al. 2009) was used to call INDELS, including large insertions, and inversions and tandem repeats. Pindel was configured using inserts sizes of 250, 500, and 2,500 bp.

### Mutations and Callable Sites

Candidate mutations were detected following a similar procedure as described by Ness et al. (2015), making use of a custom Cython script and the cyvcf2 0.11.5 package (Pedersen and Quinlan 2017) (see Data Availability). We summarize the main steps for detecting SNMs and structural variants below. Callable sites were required to have an MQ of at least 50, a threshold chosen on the basis of the distribution of its observed values. Similarly, a minimum value of the Phred quality score (QUAL) of 100 was set for all nuclear contig sites, but a lower threshold of 70 was set for the organelle genomes, based on their distribution of QUAL values. The distribution of contig read depths (DP) did not show clearly distinct peaks, and thus a minimum combined DP for all lines was set to 167 for the nuclear contigs, which is one standard deviation below their mean DP value. Similarly, this threshold was set to a combined DP of 26,000 for the plastid and 20,000 for the mitochondrial genome. In addition, only callable sites marked as haploid and with Phred-scaled genotype quality (GQ) equal to its maximum value of 99 in at least three lines were called. In order to compare the frequency of callable sites between MA lines, a line-specific callable rate was also estimated from a separate set of alignment files (one per line) containing synthetic mutations at known sites (Farlow et al. 2015; Keightley et al. 2015). These mutations were distributed every 27 kb, and called as regular mutations (see below), so a callable rate was estimated from the number of mutations retrieved relative to the total number introduced.

Since mutations are assumed to be extremely rare events, variant sites were only considered as mutation candidates when the alternate allele was present in only one line. Candidate mutation sites were further required to have a GQ of 99, be biallelic, have a minimum depth of six reads, and with no more than one in six reads containing the reference allele. The alignment context was also taken into account, by removing candidate mutations that occurred no farther than 10 base pairs from a site where at least 14 lines contained more than 1 out of 6 reads with alternate alleles. Mutation candidates were further validated by visualization of snapshots generated using the batch mode of the Integrative Genomics Viewer, IGV (Robinson et al. 2011; Thorvaldsdóttir et al. 2013).

For comparative analyses, a data set of callable sites from *C. reinhardtii* was also used. This corresponded to the one used by Ness et al. (2015), including genome-wide information for reference and mutated alleles and their position, and included more than 5,000 SNMs from approximately 95 Mb of callable positions (combined over ancestral strains). Callable and mutated sites were defined as above. Other parameters related to sequence context and annotation of genomic features were recalculated using the same tools and methods as used for *C. incerta* (see below).

### The Mutation Rate and Its Distribution

The mutation rate (*μ*) was estimated as *μ = Nμ/(Nc × Nlines × t)*, where *Nμ* is the number of mutations found, *Nc* the length of callable genome (in base pairs), *Nlines* is the number of MA lines, and *t* the number of generations. When *μ* was estimated for individual lines, contigs or genomic features, the numerator and denominator were adjusted accordingly. The distribution of SNMs among lines was fitted to parametric distributions using the R package fitdistrplus 1.1-1 (Delignette-Muller and Dutang 2015). Normal, log-normal, gamma, Poisson, and exponential distributions were tested, and the one with the lowest value of the Akaike information criterion was selected. The Poisson distribution was chosen as a null hypothesis for the expected variance of the number of mutations among lines (Charlesworth 2012). This variation was also evaluated by simulating the MA experiment using SLiM 3 (Haller and Messer 2019). The callable genome length and mutation rate of *C. incerta* were calculated assuming a haploid, neutral model with no recombination. A total of 1,000 replicates were simulated for the estimated average number of generations of the experiment (788 generations).

MA line 2 was excluded when computing mutation rates, as it was found to share an unusually high number of alternate alleles with MA line 3 (75%, compared with the mean 1% shared with the remaining lines), suggesting possible contamination during the maintenance or sequencing of the lines. A neighbor-joining dendrogram generated using vcf-kit phylo (Cook and Andersen 2017) illustrates this genetic relationship (supplementary fig. S13, Supplementary Material online). Two SNMs found uniquely in MA line 2 were included for all remaining analyses.

Counts of SNMs causing synonymous and nonsynonymous changes within coding sequences were used to estimate the *Ka/Ks* ratio (Hurst 2002) and to test for the possible existence of selection during the MA experiment. Since only one or a few mutations are expected within each coding sequence, all coding sequences with at least one SNM were concatenated. The processing of the files was done using the bedtools v2.29.2 intersect and getfasta utilities (Quinlan and Hall 2010), and the *Ka/Ks* ratio was estimated with KaKs_Calculator 2.0 (Wang et al. 2010). Amino acid mutation effect types were also classified as low (synonymous), moderate (missense), or high (stop lost/gain) using SnpEff 4.3t (Cingolani et al. 2012), and their functional annotation was obtained both with biomaRt 2.44.0 (Durinck et al. 2005, 2009) using the Phytozome database (Goodstein et al. 2012), and BLAST (Boratyn et al. 2013) using the standard nucleotide database.

The IMD was used as an indication of the level of clustering of mutations. This parameter was estimated by measuring the distance (in base pairs) between SNMs belonging to the same contig, irrespective of the MA line carrying it. To determine whether the observed IMD was distributed differently from a random expectation, the IMD was also computed for the same number of mutations, located at random callable sites. This process was repeated for 1,000 iterations. Mutation clustering was also measured by counting the number of SNMs in genomic sections of 1, 10, 100, and 500 kb, and numbers were compared with the expected number of mutations in these sections, following the same procedure as described above.

### Genomic Context

The genomic context of the mutations was explored in two ways, first by means of parameters that collapse genomic information into simple parameters and second by identifying sequence motifs and features.

Local genomic information was first summarized by measuring GC content, but other parameters such as Shannon Entropy (*E*) and linguistic complexity (*L*) were also calculated (Schneider 2000; Vinga 2014). These parameters were computed in windows of 2, 10, 100, and 1,000 bp extending upstream and downstream from a mutation site, including the mutation site. Genomic intervals and GC content were measured using the bedtools makewindows and nuc utilities. *E* and *L* were measured using NeSSie (Berselli et al. 2018). Variability in GC, *E*, and *L*, measured as the standard deviations of these parameters within windows overlapping a given position were also computed and then averaged for windows of 5, 21, 201, and 2,001 bp (obtained as above).

Regarding sequence motifs, we considered the trinucleotide context of every site. For a given site, three different contexts were obtained: upstream, downstream, and surrounding contexts, all based on trinucleotide sequences, including the site whose context was extracted. For example, taking the sequence AGATA, where the middle and underlined A is the reference site whose context is of interest, three different contexts are obtained: AGA (upstream context), GAT (surrounding context), and ATA (downstream context). In order to reduce the total number of possible context sequences, trinucleotide context sequences were edited so that the reference site was always either A or C. For example, given the sequence TATCT, the surrounding context of the underlined T, ATC, would we converted to GAT using the reverse complement. The upstream and downstream contexts were also deduced using the reverse complementary sequence, but including a swap between these contexts, for example the upstream context TAT would be considered as downstream context using the reverse complement: ATA.

In addition, annotations for different genomic features were used to provide genomic context. Gene annotations for *C. incerta* were obtained from Craig et al. (2021) and the *C. reinhardtii* v5.6 annotation (Merchant et al. 2007) was obtained from Phytozome. We limited the analyses to coding sequences, introns, UTRs, and intergenic regions. When features overlapped, coding sequences took precedence over other site classes, followed by 5ʹ and 3ʹUTRs, and finally introns. Repetitive sequences were identified by providing RepeatMasker v4.0.3 (Smit et al. 2013–2015) with a custom repeat library containing manually curated transposable elements (TEs) from *C. reinhardtii*, *C. incerta*, *Chlamydomonas schloesseri, Edaphochlamys debaryana*, and *Volvox carteri* (see Craig et al. [2021]). Repeats were classified as TEs, low complexity regions (defined as single nucleotide repeats), and microsatellites. TEs were further divided by class (i.e., class I and II) and order/superfamily following Wicker et al. (2007). Distance (in base pairs) was also calculated from each genomic feature to the closest mutation site.

### Modeling Mutability

Generalized linear models allow the estimation of the effects of genomic factors on mutability based on data sets containing sites classified either as mutated or nonmutated. Here, we used a regularized regression approach that introduced a penalization factor to constrain the magnitude of the coefficient estimates, which reduces the correlation between the variables used as predictors in the model. A database was first built for all callable genomic positions. Only presence/absence of nuclear SNMs was evaluated as the response variable in the model. As predictor variables, we included variables related to genomic context, including GC, *E*, and *L* content in different genomic window sizes, trinucleotide motifs, genomic features, and distances, as detailed above. Positions where any of these variables were missing were removed from the data set, along with near-zero variance predictors. All factors were converted into dummy numerical variables, centered, and scaled so that a unit change corresponded to a change of one standard deviation. Given the large number of parameters, and the likely correlation among them, a logistic regression model was run via penalized maximum likelihood making use of elastic net regularization using the glmnet R package 4.0-2 (Friedman et al. 2010). This allows first for optimizing penalization factors using a 10-fold cross-validation method, where 75% of data are used for training and 25% for validation. The generalized model was then run in a similar fashion as described in Ness et al. (2015), using a training set composed of all the detected mutations and 10^5^ random nonmutated sites. The performance of the model was tested against a larger data set containing all mutated sites and 10^6^ nonmutated random callable positions. In addition, fits and predictions were run ten times, using independent training and test sets, including model prediction across species (see below). Confidence intervals were built for the predicted response and regression coefficient estimates based on their variation over replicates.

A second set of models was fitted in order to model the occurrence of the six different types of SNM (A→C, A→G, A→T, C→A, C→G, and C→T). The data set used here was obtained from the previous one with the following changes. First, only mutation sites were considered in the data set. Variables related to GC content were excluded, and those related to trinucleotide context were edited so that the mutated site was excluded from them. The data set was split in two, one for training containing 75% of the observations, and an independent data set with the remaining 25% for testing. Given the multinomial nature of the response variable, we trained different classification and regression models using the caret package v6.0-86 (Kuhn 2020), including penalized regression as described above (glmnet), but also including machine learning methods based on regularized random forests and neural networks (nnet). Since the different SNM types were unevenly represented in our data set (e.g., C→T mutations were largely over-represented), different strategies to correct data imbalance were considered, including random down-sampling and up-sampling, and the synthetic minority over-sampling technique, SMOTE (López et al. 2013). In addition, a model was run where the response variable only had two levels, C→T mutations (the most abundant SNM type), and other SNM types grouped together. The same training set was used in each case, by setting the same seed for sampling random numbers. An R environment (version 3.6.1, R Core Team 2020) was used for all statistical analyses, including prediction of mutation properties. Predictive models were also run on a genomic data set with *C. reinhardtii* data from Ness et al. (2015), and cross predictions were performed using one species’ data set for training, and the other’s for testing (e.g., by fitting the model with *C. incerta* data and making predictions on *C. reinhardtii* data).

## Supplementary Material

Supplementary data are available at *Molecular Biology and Evolution* online.

## Supplementary Material

msab140_Supplementary_Data
