# Supplementary material for: De Novo Mutation Rate Variation and Its Determinants in Chlamydomonas
Source: Mol Biol Evol. 2021 May 5;38(9):3709–23. doi: 10.1093/molbev/msab140 (PMC8383909; doi:10.1093/molbev/msab140)
Supplement: msab140_Supplementary_Data [file msab140_supplementary_data.zip › Supplemental Figures.pdf]

Supplemental Figures

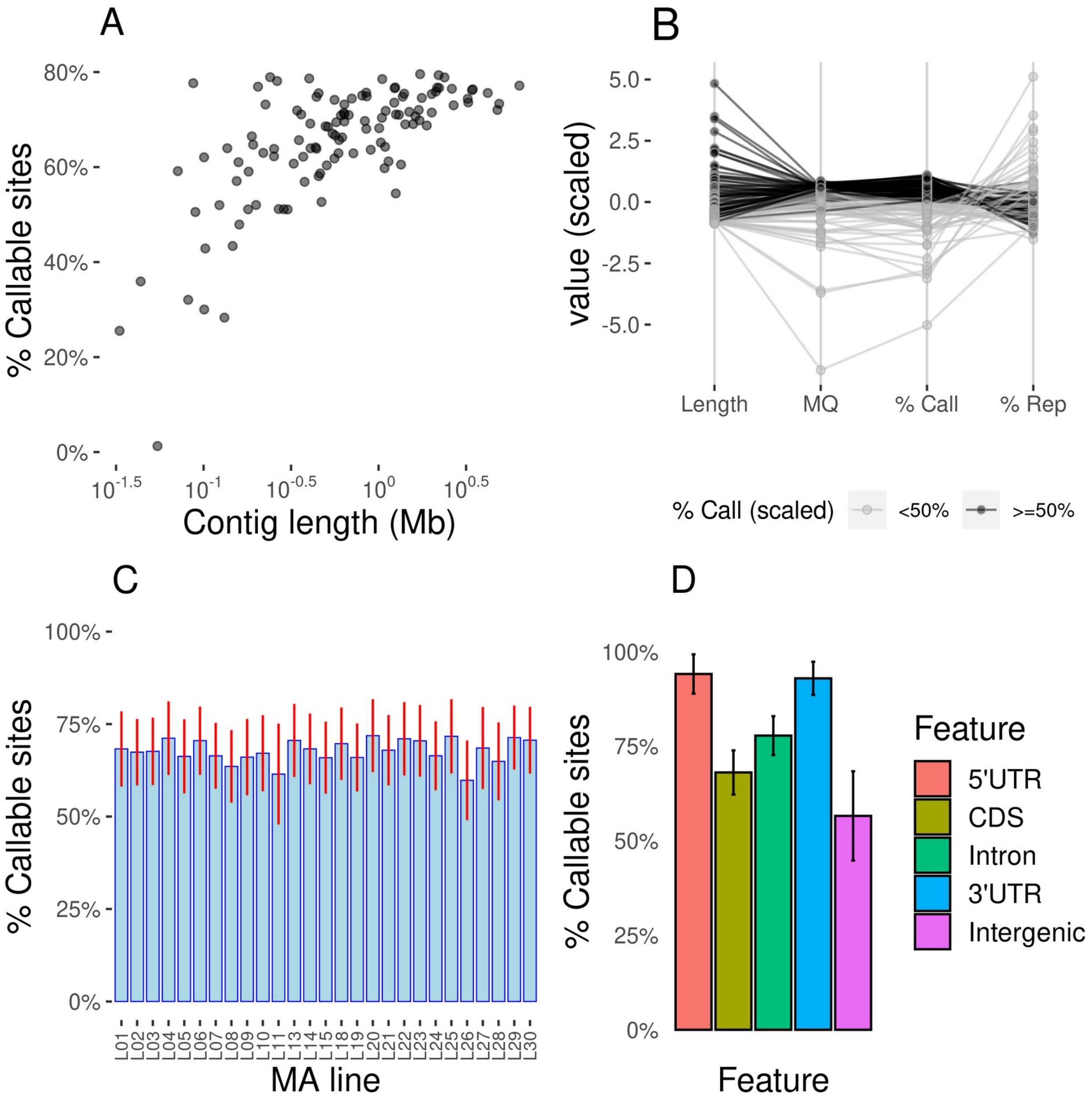

Figure S1. Callable rate in the *C. incerta* nuclear genome. A) Proportion of callable sites by contigs of different length. B) Parallel coordinates showing the relationship between contig length, mapping quality (MQ), proportion of callable sites (% Call) and proportion of repetitive sequence (% Rep, including low complexity, microsatellite and TE sequences) for the 50% less callable (grey) and more (black) callable contigs. All values are scaled to standard deviations. C) Proportion of callable sites for synthetic mutations in different MA lines. Red error bars show standard deviation between contigs of the same MA line. D) Proportion of callable sites by gene-related feature. Error bars correspond to standard deviations between contigs.

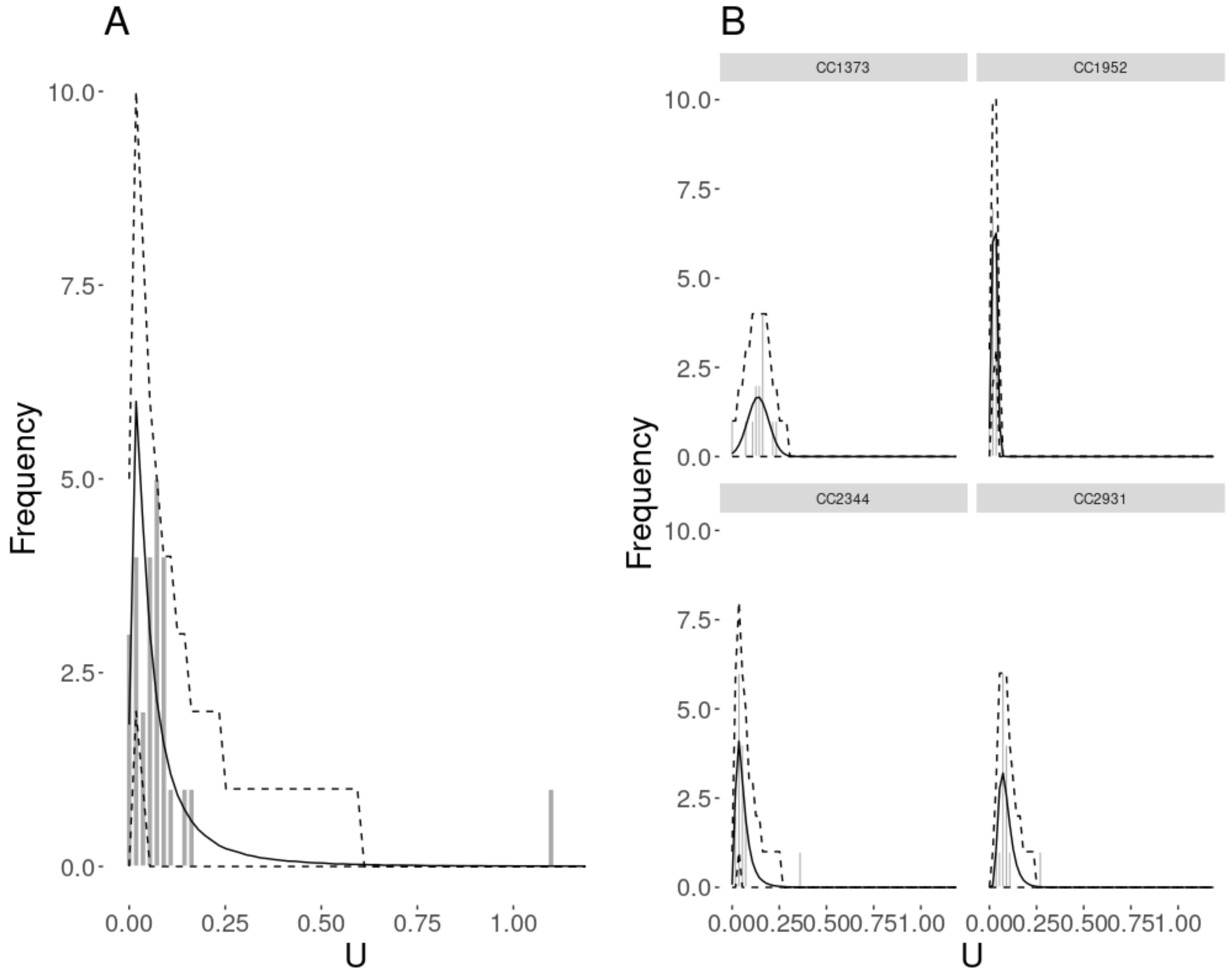

<u>Figure S2.</u> Distribution of the genomic-wide mutation rate (U), computed as the number of SNMs in the MA lines divided by the number of generations of the MA experiment. Bars correspond to the observed distribution. Solid lines correspond to the expected distribution sampled from parametric distributions, and dashed lines to its 95% confidence interval, based on 10^4 iterations of sampling. In each case, the samping distribution with the best fit following the Akaike information criterion was chosen among the following: exponential, gamma, lognormal, normal, and Poisson. A) Distribution for the *C. incerta* MA lines, fitting a lognormal distribution (meanlog = -3.01, sdlog = 1.15, KS test, P = 0.60). B) Distribution for *C. reinhardtii* MA lines derived from four representative ancestral strains (CC1373, CC1952, CC2344 and CC2931), using the data from Ness et al. (2015). Distribution parameters for these strains are: CC1373, Normal (mean = 0.14, sd = 5.56×10^{-2}, KS test, P = 0.88), CC1952, Normal (mean = 2.74×10^{-2}, sd = 1.17×10^{-2}, KS test, P ≈ 1), CC2344, Lognormal (meanlog = -3.03, sdlog = 0.65, KS test, P = 0.49), CC2931, Lognormal (meanlog = -2.52, sdlog = 0.41, KS test, P = 0.51).

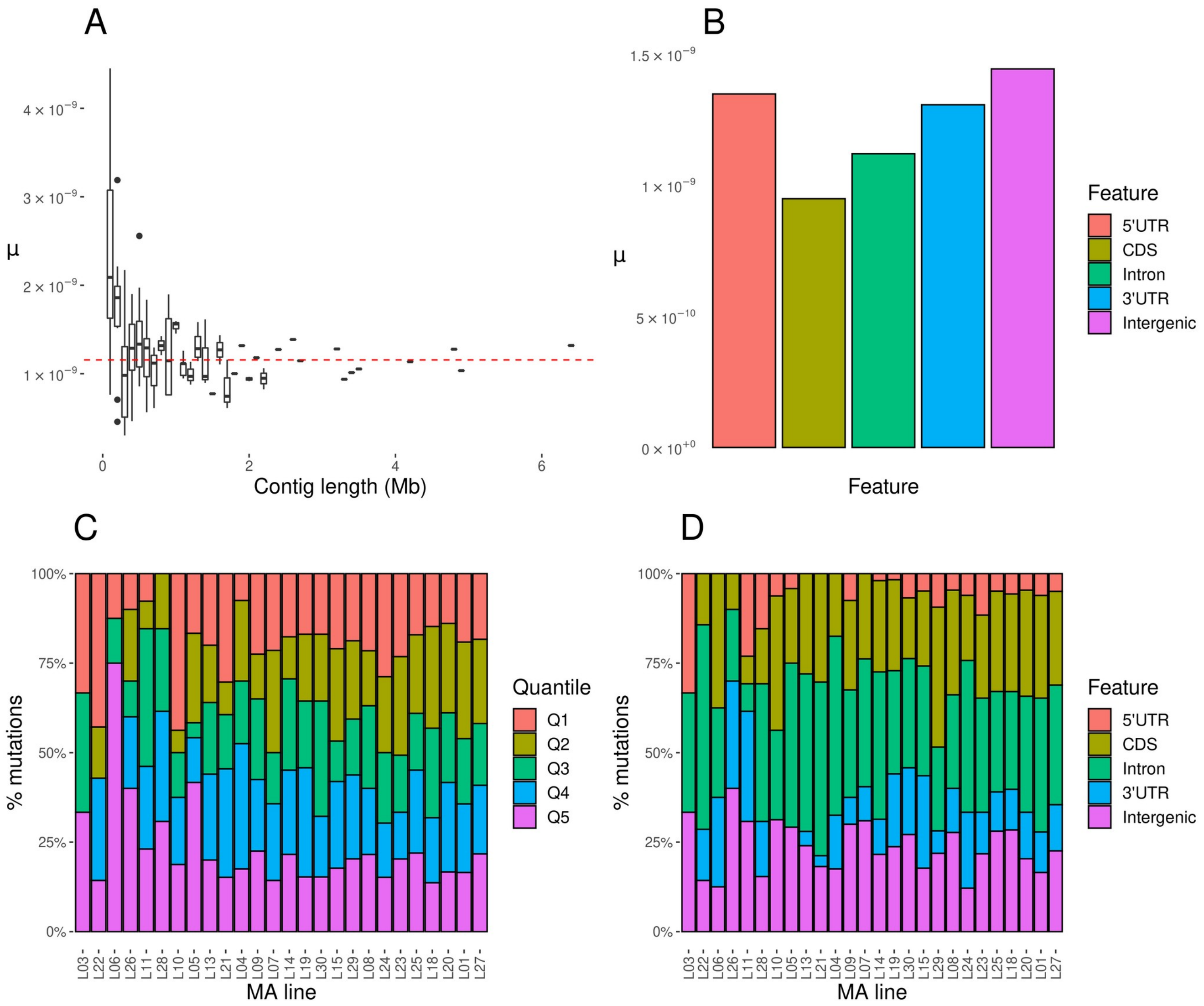

Figure S3. Distribution of mutations over contigs and genetic features. A) Variation in mutation rate (μ) with contig length in megabases (Mb). Contigs were binned in 0.1 Mb steps according to their length. The red dashed line indicates the overall mutation rate (μ = 8.78 x 10^{-10}). B) Mutation rate over different genetic features. C) Distribution of the proportion of mutations per contig grouped by length and MA line. The cumulative length of contigs sorted by size was used to build 5-quantile thresholds (e.g. Q1 represents contigs with a cumulative length equal to 20 % of all genomic callable sites). MA lines are sorted from lower (left) to higher μ (right). D) Distribution of the proportion of mutations at different genentic features for each MA line. MA lines are sorted from lower (left) to higher μ (right).

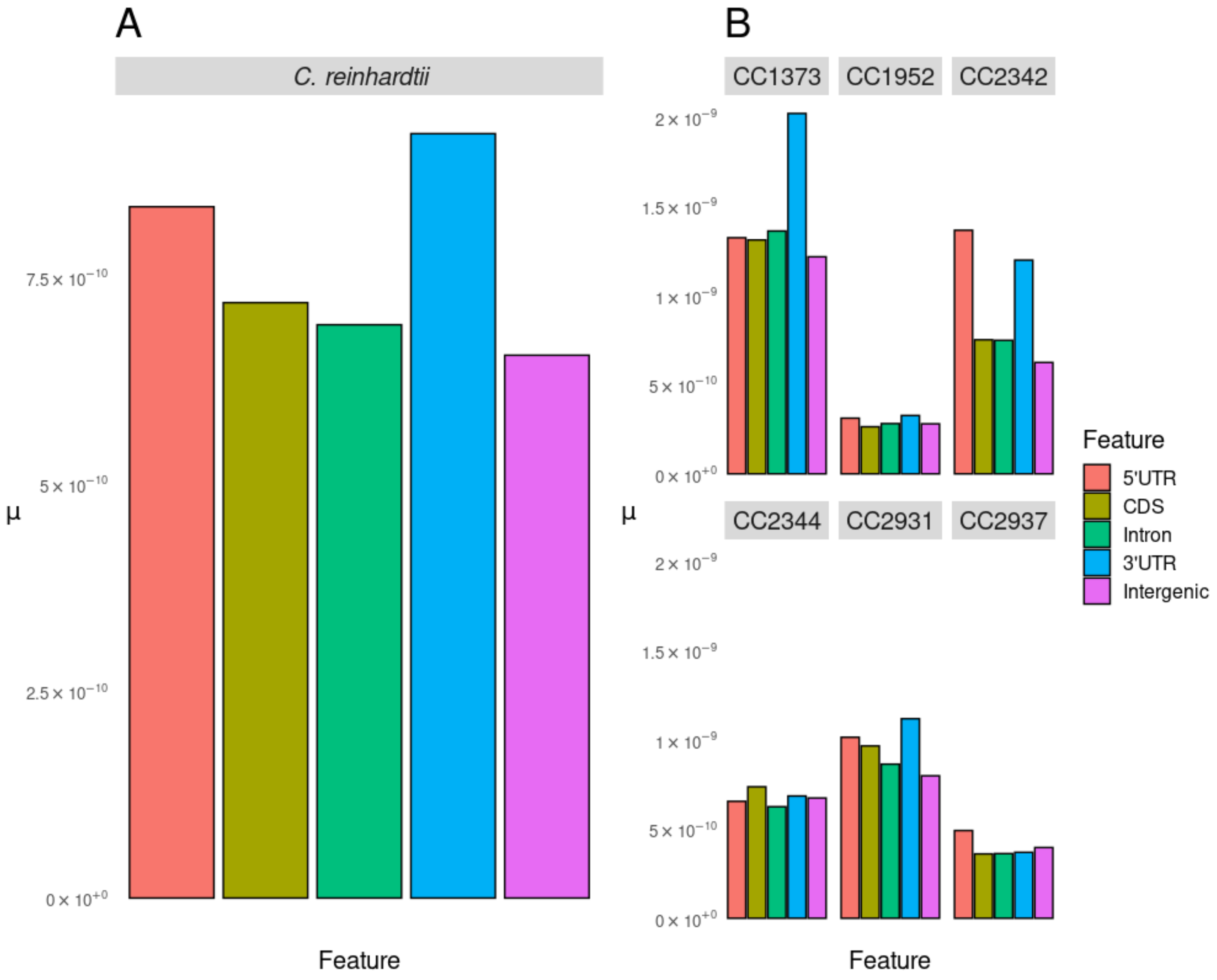

Figure S4. Distribution of mutations over genetic features in *C. reinhardtii*. A) Distribution using mutations from all MA lines and strains available in Ness et al. (2015). B) Distribution for each of the *C. reinhardtii* strains analysed (CC1373, CC1952, CC2342, CC2344, CC2931, CC2937).

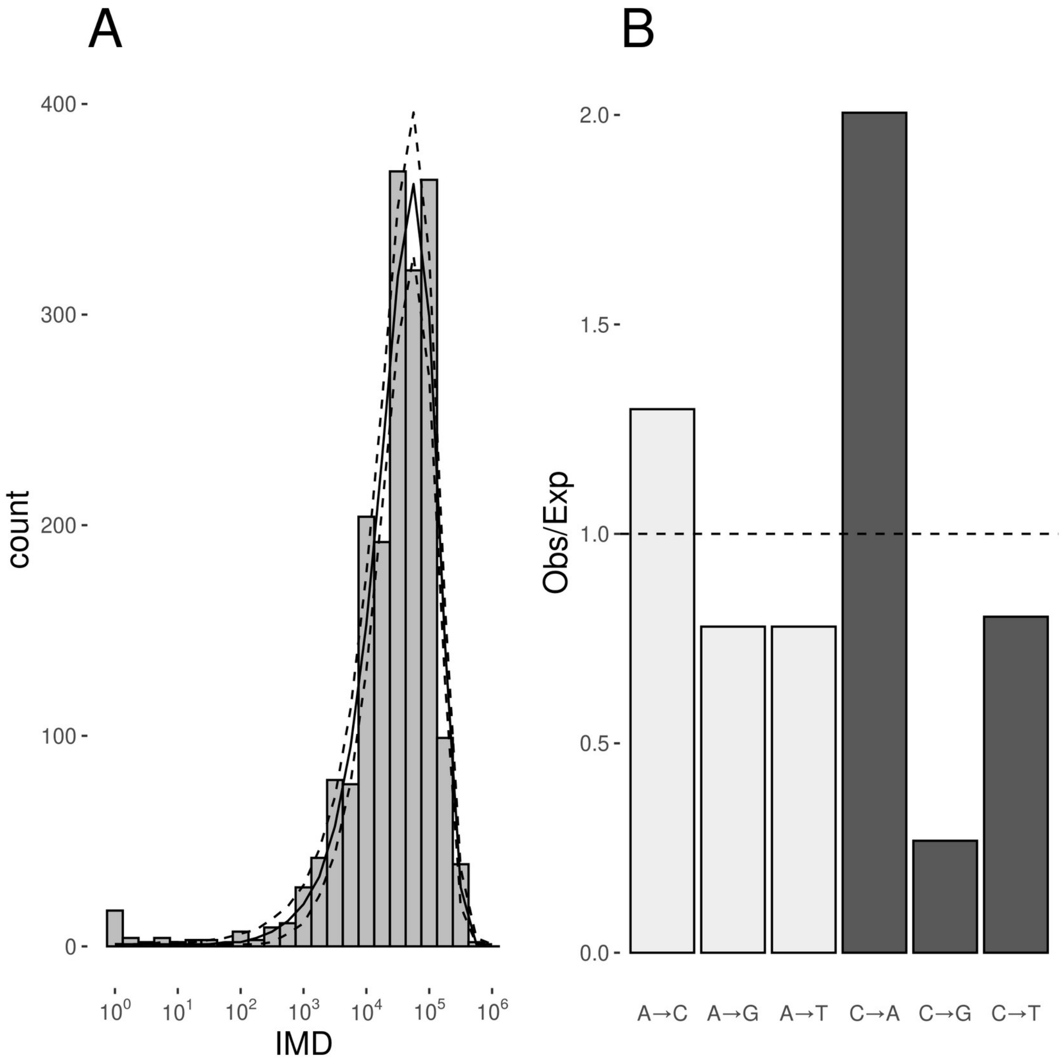

Figure S5. Clustered mutations. A) Distribution of inter-mutation distances (IMD). The observed distribution is shown with bars, and the expected distribution as a solid line (95% confidence intervals in dashed lines). B) Spectrum of single nucleotide mutations (SNM) that were adjacent to other SNMs. The height of the bars relates to the deviation of the observed distribution from the expected at random after correcting by GC content. Light bars refer to mutations at A:T sites, while dark bars refer to C:G sites.

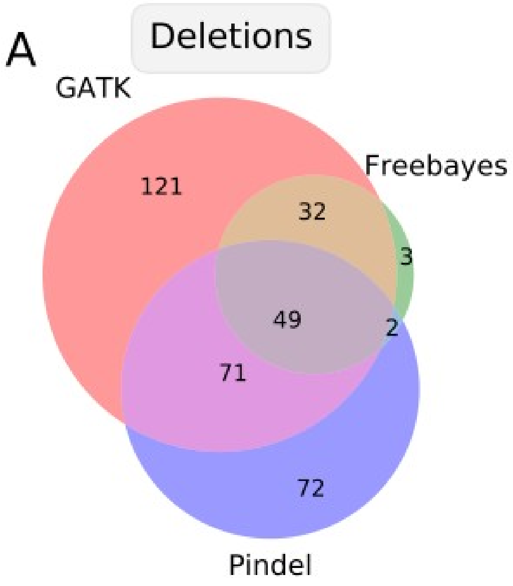

<u>Figure S6</u>. Venn diagrams showing the number of A) deletions and B) insertions discovered using three different software packages: GATK v4.1.4.0 (red), Freebayes 1.3.2 (green) and Pindel v0.2.5b9 (blue).

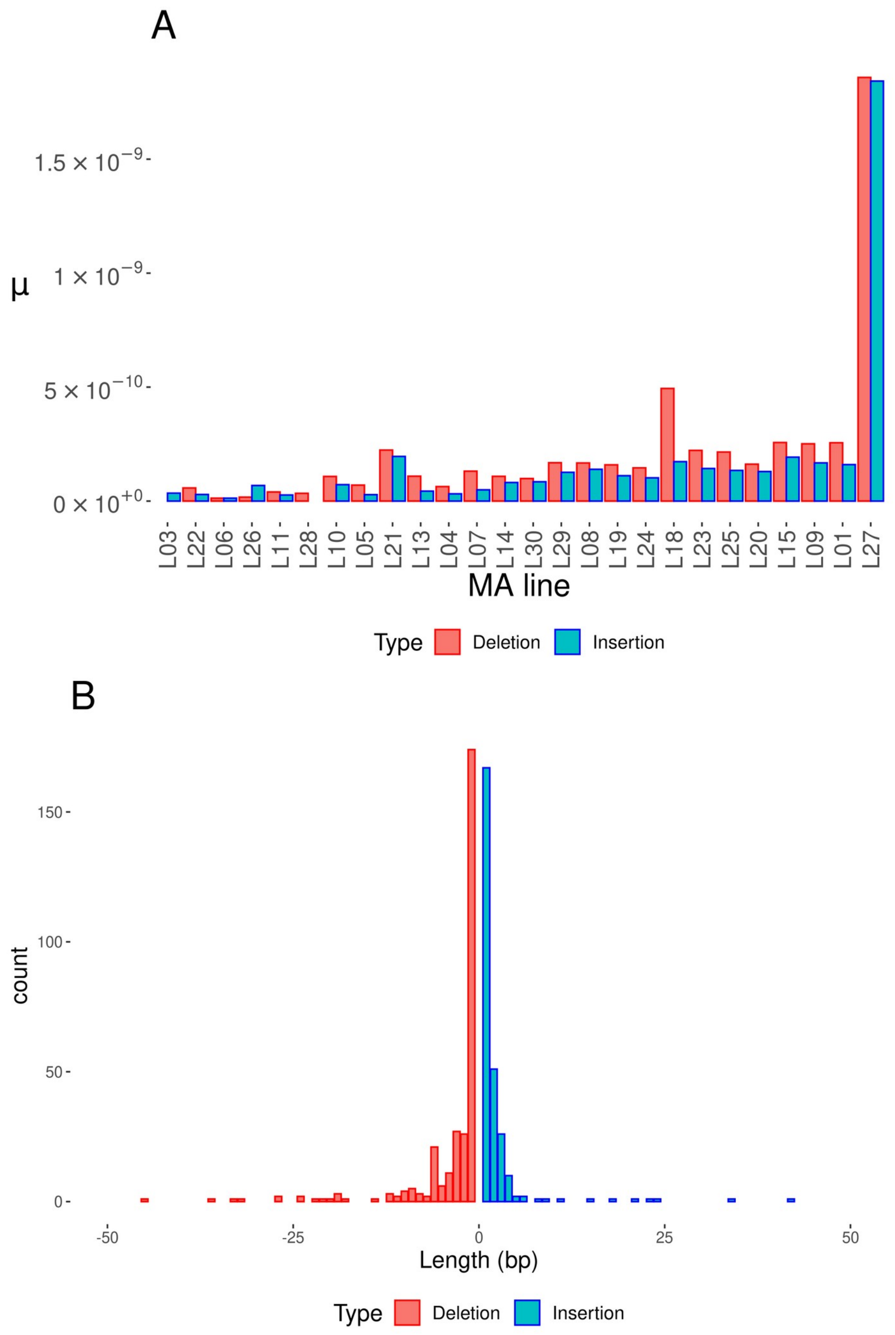

Figure S7. INDEL distribution. A) Distributon of the mutation rate (μ) for deletions (red) and insertions (blue) across MA lines. Lines are sorted from lowest SNM mutation rate (left) to highest mutation rate (right) as in Figure 1 of the main text. B) Distribution of the number of deletions (red) and insertions (blue) by the their length (in base pairs). Only INDELS shorter than 50 bp are shown.

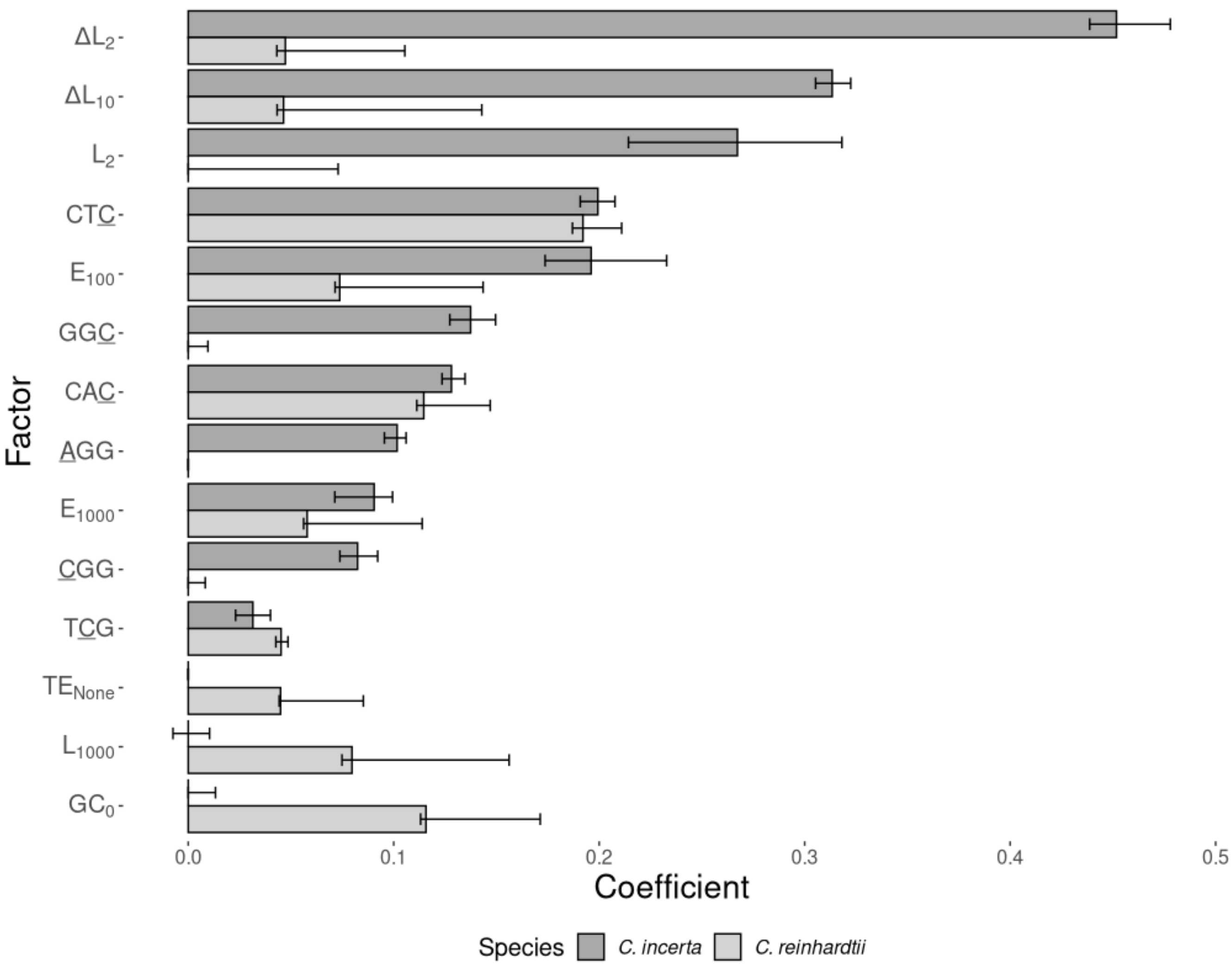

Figure S8. Regression coefficient estimates for the predictive model on genomic mutability. Estimates correspond to ten training set replicates of *C. incerta* (dark bars) and *C. reinhardtii* (light bars). Only the ten most important factors found in either *C. incerta* or *C. reinhardtii* are shown. Factors included in the figure are: Variation in sequence repetitiveness (ΔL_2 and ΔL_{10}, measured in windows of 2 bp and 10 bp respectively extending downstream and upstream), mean sequence repetitiveness (L_2 and L_{1000}, measured in a window of 2 bp and 1000 bp respectively extending downstream and upstream), nucleotide repetitiveness (E_{100} and E_{1000}, measured in a window of 100 bp and 1000 bp respectively extending downstream and upstream), GC_0 (GC content at the reference site), TE_{None} (sites with no transposon annotation) and CTC, CAC, GGC, CGG, AGG and TCG trincucleotides (the underlined C or A being the reference site). Factors are sorted by their estimated effect on *C. incerta*. All predictors were standardized prior to regression, so their effect sizes are comparable.

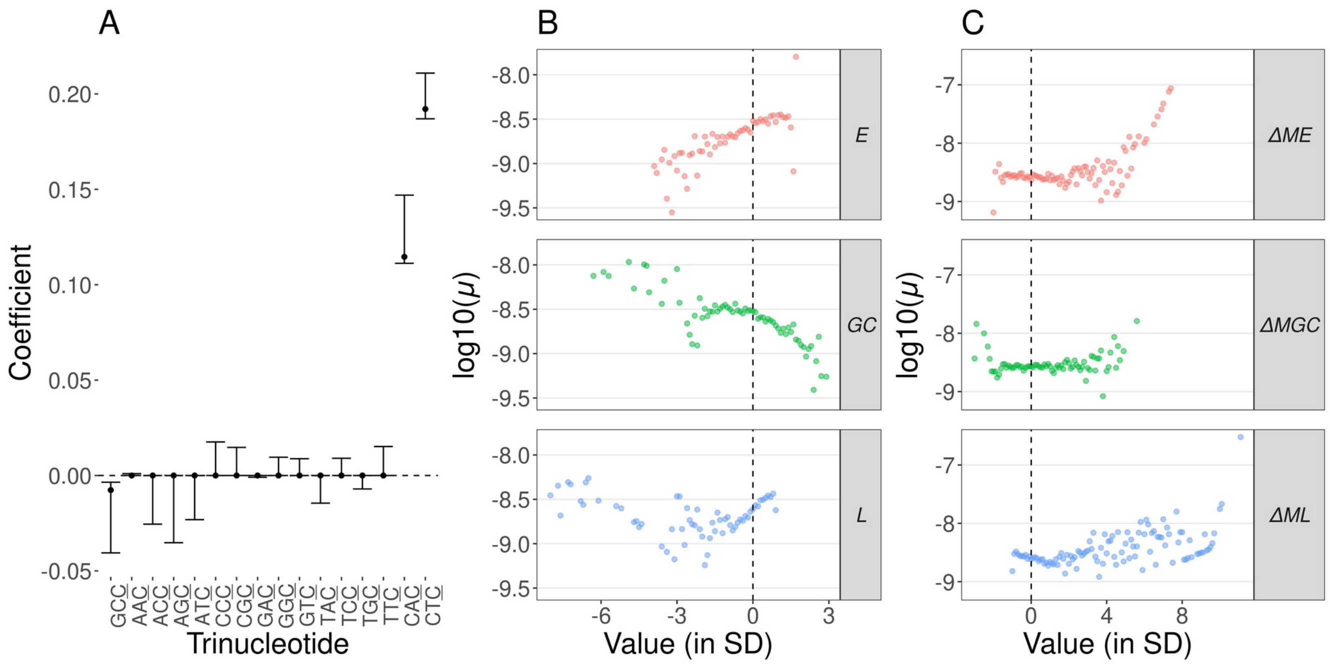

<u>Figure S9.</u> Relationships between sequence context, base composition, sequence complexity and mutability in *C. reinhardtii*. A) Regression coefficient estimates for the 16 possible dinucleotides upstream of reference C sites. B) Relationship between scaled mean nucleotide repetitiveness (*E*), GC content (*GC*) and sequence repetitiveness (*L*), measured in genomic windows of 2 Kb. C) Relationship between the scaled variation in nucleotide repetitiveness (*ΔE*), variation in GC content (*ΔGC*) and variation in sequence repetitiveness (*ΔL*), measured in a genomic windows of 20 bp. Note that standard deviations are used as the unit of measurement for the genomic parameters in plots B) and C). Only genomic sections where $\mu > 0$ are shown in B) and C).

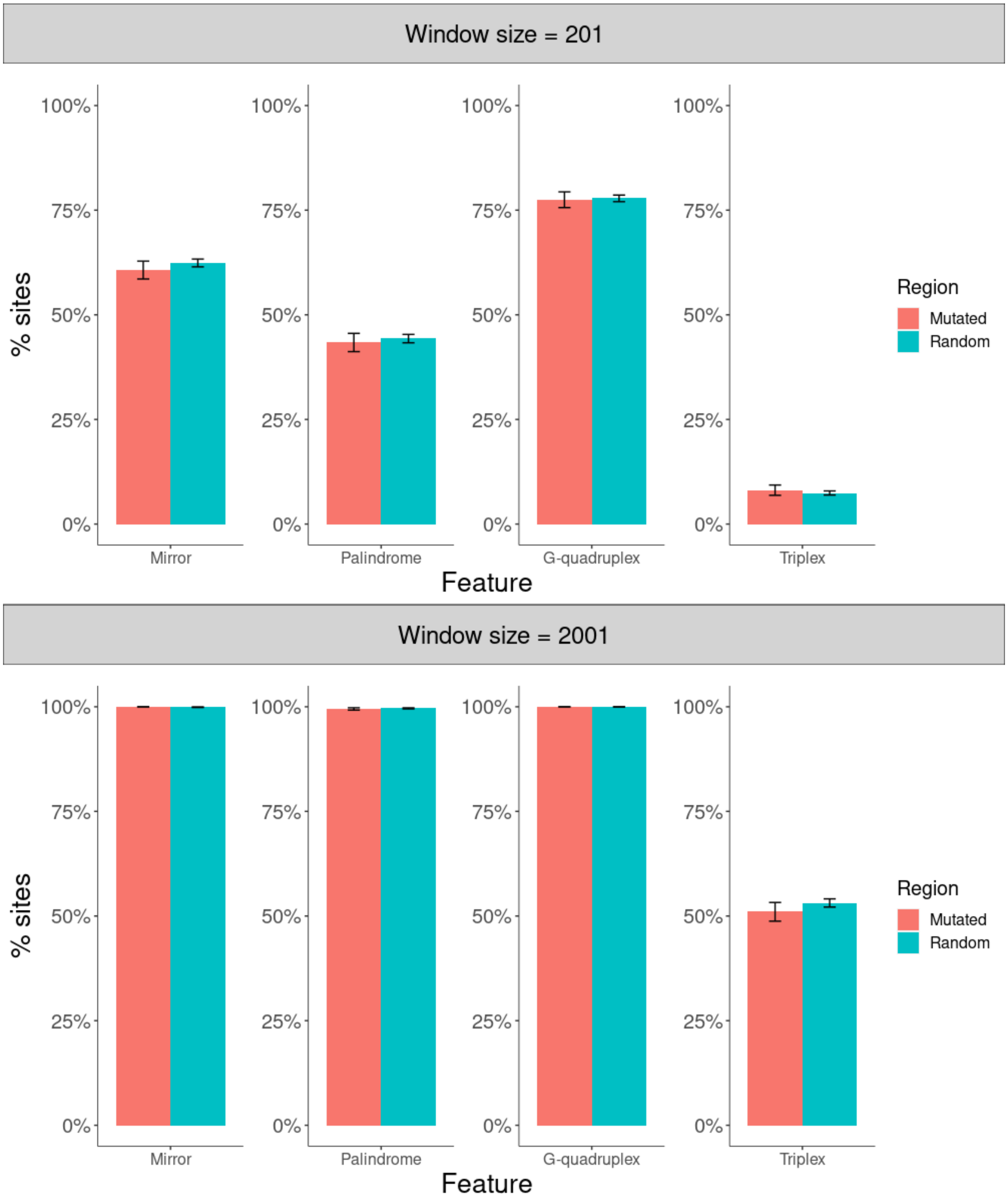

Figure S10. Percentage of genomic sites containing at least one of the following features: mirror, palindrome, G-quadruplex, and triplex in a windows of 201 (upper panel) or 2001 (lower panel) bp. Regions are grouped into those containing known mutated sites, containing a total of 1,991 SNMs (in red), and 10^4 randomly sampled genomic locations (in blue). Significance is calculated using the Kruskal-Wallis test (* $P < 0.05$, ** $P < 0.01$, *** $P < 0.001$). Confidence intervals (95 %) are based on 1000 bootstrap replicates.

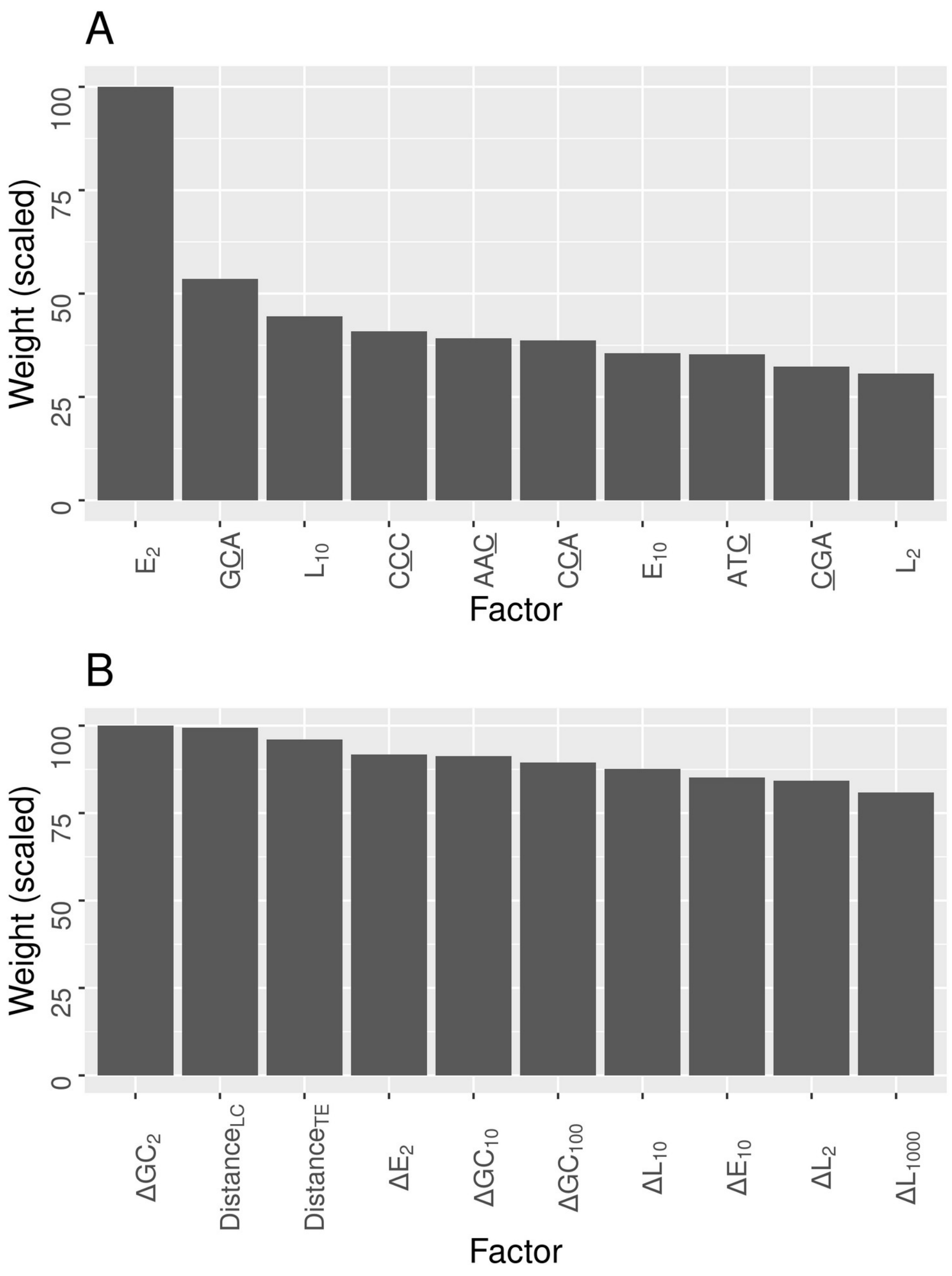

Figure S11. Scaled weight (from 0 to 100) for the predictor used in two classifying machine learning algorithms: A) Upsampled neural networks (using *C. incerta* data), and B) upsampled regularized random forest (using *C. reinhardtii* data). The choice of methods is based on those that produced the highest accuracy in each species. Only the ten factors with the highest rank are shown. Factors are measurements of GC content, nucleotide (*E*) or sequence (*L*) repetitiveness and their variation (prefixed with Δ) in a window size of 2, 10, 100 or 1000 bp extending downstream and upstream, trinucleotide context (the underlined C being the reference site), and distance to low complexity (LC) or transposon elements (TE).

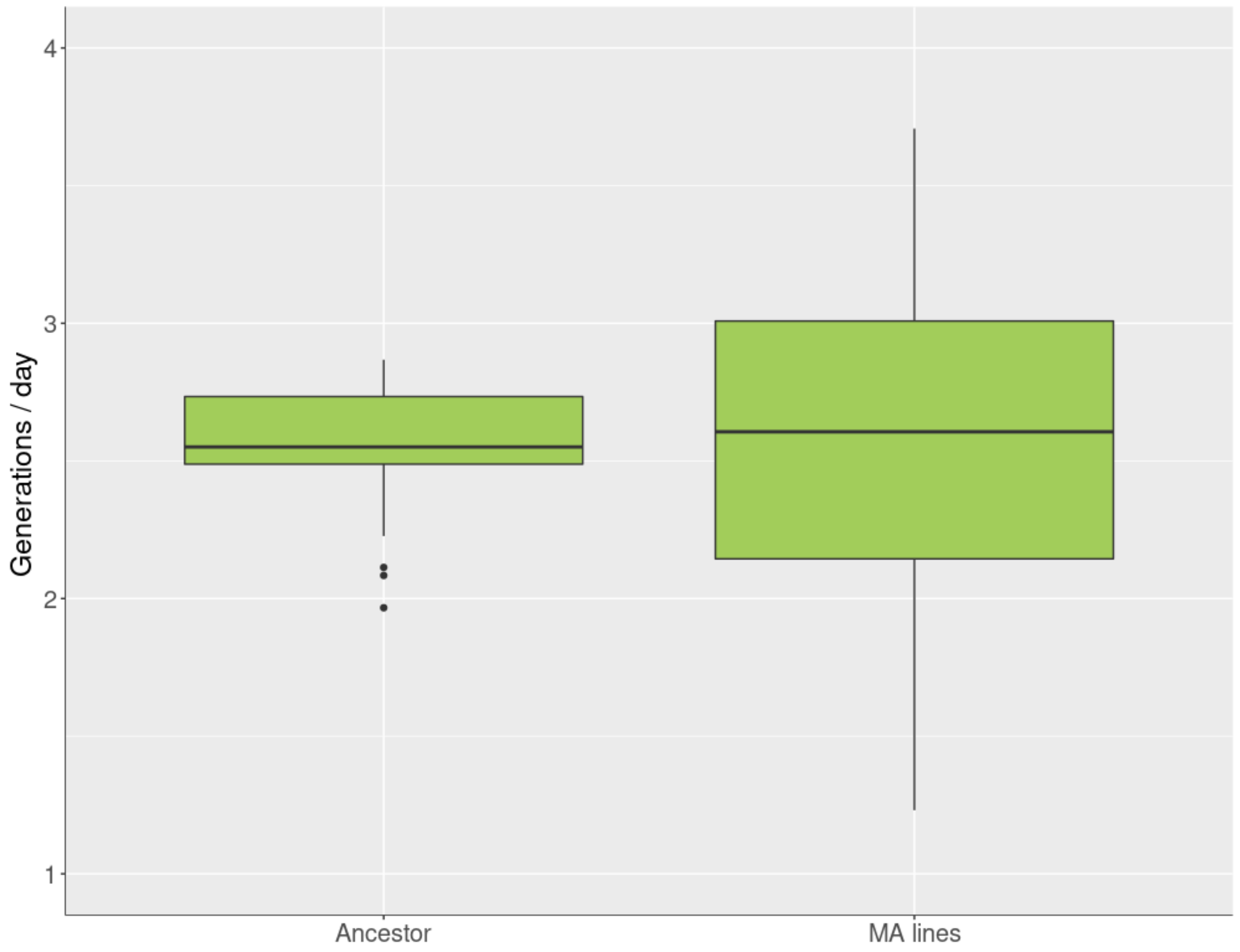

<u>Figure S12.</u> Average number of generations per day for replicates of the *C. incerta* ancestor strain SAG 7.73 (left), and for 14 *C. incerta* MA lines. The experiment to estimate generation times was performed at the end of the MA experiment.

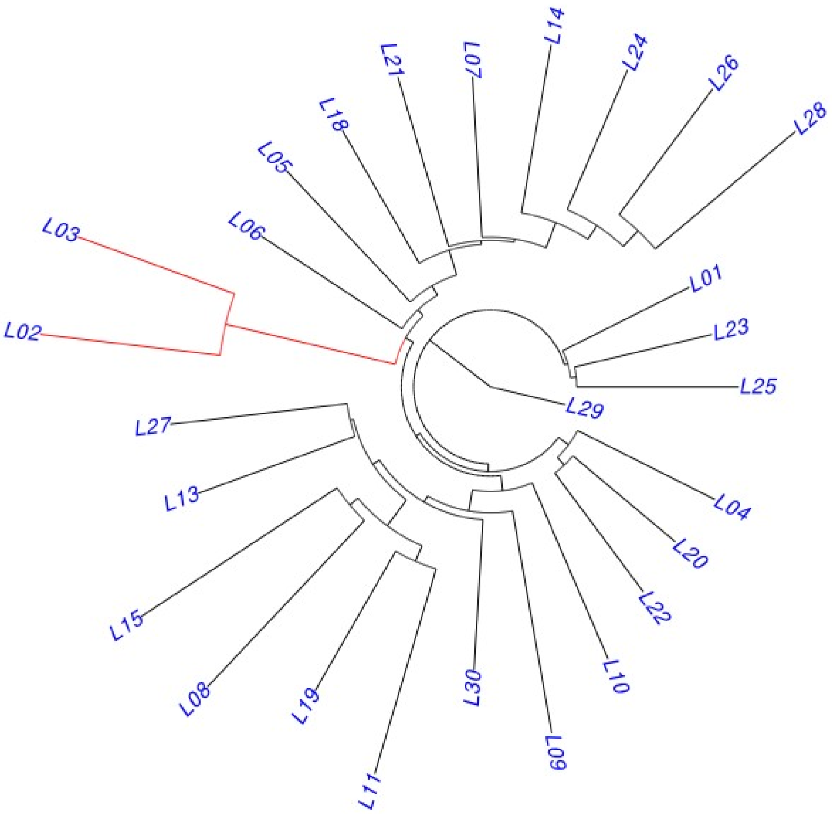

Figure S13. Unrooted neighbor-joining dendrogram showing genetic relationships of the 27 MA lines based on their genotype for 885 shared alternate alleles at callable positions (excluding de novo mutations and indel variants). The common branch for MA lines L2 and L3 is colored in red. Note the tree does not aim to represent true phylogenetic relationships, since the MA lines were derived independently from a common ancestor, but to highlight the higher genetic relationship of MA lines L2 and L3, which is probably due to contamination.

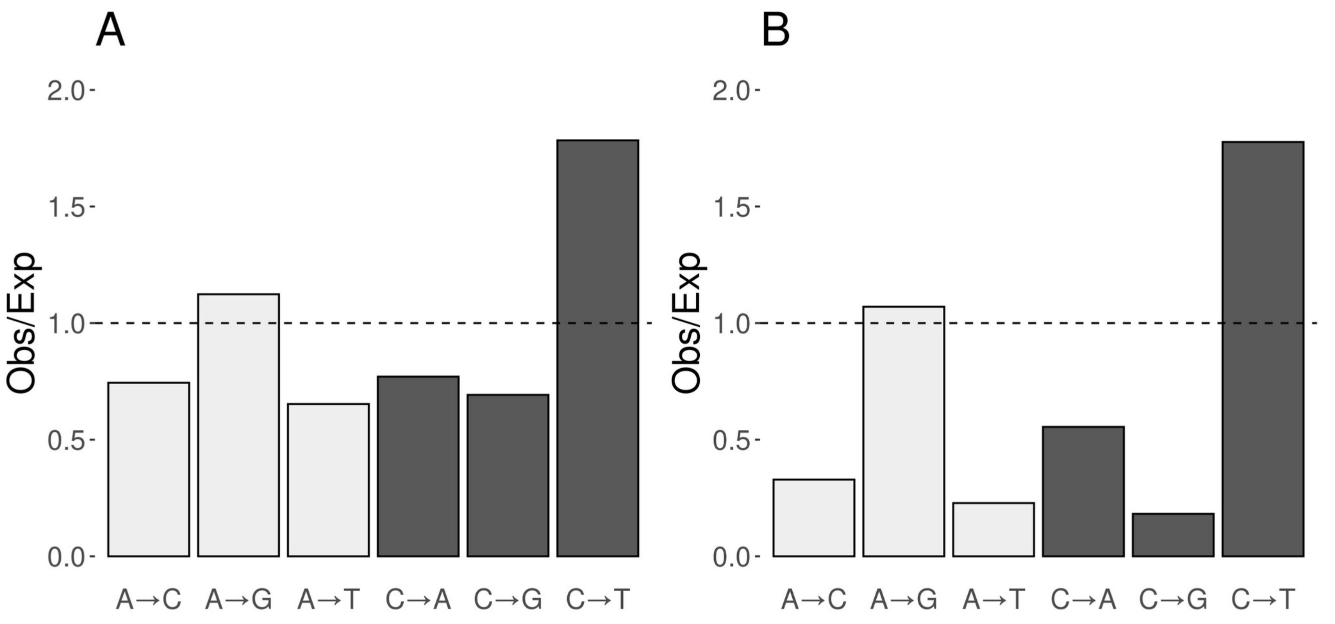

Figure 14. Spectrum of SNMs in the MA lines excluding the hypermutant L27 (A), and in L27 only (B). Light bars refer to mutations at A:T sites whereas dark bars refer to C:G sites. The height of the bars relates to the deviation of the observed distribution from the expected values. In A) the expected values are based on all SNMs being equally represented after accounting for GC content. In B) the expected values are based on the SNM spectrum shown in A).
