# Supplementary material for: De Novo Mutation Rate Variation and Its Determinants in Chlamydomonas
Source: Mol Biol Evol. 2021 May 5;38(9):3709–23. doi: 10.1093/molbev/msab140 (PMC8383909; doi:10.1093/molbev/msab140)
Supplement: msab140_Supplementary_Data [file msab140_supplementary_data.zip › File S1.pdf]

Mutation rate variability and hypermutant line 27

Mutation accumulation (MA) line 27 (L27) was found to harbour nearly 42% of all the *de novo* mutations discovered in this study (μ = 131.7 x 10^{-10}), and its mutation rate was at least 75% higher than that of *C. reinhardtii* hypermutant MA lines described by Ness et al. 2015. With a total of 829 single nucleotide mutations (SNMs), its SNM rate per haploid genome approximates to $U \approx 1.11$, which is close to that observed for RNA viruses (Drake et al. 1998). Deletions (μ = 18.6 x 10^{-10}) and insertions (μ = 18.4 x 10^{-10}) were also overrepresented in L27 compared to the other MA lines, in line with the positive correlations observed between SNM and insertion and deletion rates among MA lines (Figure 1b-c). No inversions or large deletions (> 50 bp) were observed in L27. The proportions of mutations per genomic feature (e.g. coding region) and per contig grouped by length were similar in L27 and the other lines (Figures S3c-d). After accounting for GC content, C→T mutations were more than 3-fold more frequent in L27 than expected, and 2-fold more frequent in L27 than expected, given the SNM spectrum observed in the remaining lines (Figure S14).

The high mutation rate of L27 raises the question of its fitness and viability, given that most mutations are expected to be deleterious (Keightley and Lynch 2003). Previous laboratory experiments in *C. reinhardtii* have shown that fitness is negatively correlated with the mutation load, and MA lines were generally less fit than their ancestors (Kraemer et al. 2017). However, in a recent study of more than 1,500 recombinant MA lines of *C. reinhardtii* produced by back-crossing to an ancestor of the same genetic background, the average effects of individual deleterious and advantageous mutations on fitness (growth rate) were inferred to be of approximately of the same magnitude (Böndel et al. 2019). Although in the same experiment deleterious mutants were more frequent than advantageous ones, the frequency of the latter was surprisingly high (0.071 vs 0.048).

In nature, hypermutators have mostly been found in bacteria deficient for mismatch repair proteins (Mehta et al. 2019; Veschetti et al. 2020). However, we did not find mutations in coding regions of genes annotated with DNA repair functions in L27. Several missense mutations were found in genes related to ribosomal function, histone H2B and RNA polymerases I and III (Table S5), but we did not confirm their potential role in affecting the mutation rate with our study. It can be hypothesized that L27's high mutation rate could be the result of mutations arising early during the first generations of the MA experiment resulting in one or more unknown mutators.

Finally, it is also noteworthy that the mutation rate in bacteria has been found to increase by 10- to 100-fold in response to phage infection (Pal et al. 2007). A similar observation has not been made yet in eukaryotes, but should not be excluded. Fungi defense mechanisms against transposable elements (TEs) have also been described to involve repeat-induced point (RIP) mutations (Galagan and Selker 2004). Interestingly, this RIP mechanism generates C→T mutations, possibly via deamination of methylated cytosines (Clutterbuck 2011). We further investigated whether an excess of C→T mutations in MA line 27 could be attributed to TE activity. The proportion of mutations found in TE-related annotations was only slightly higher in L27 than the expectation based on the frequency observed in other lines (χ^2, P = 0.049), and the proportion of C→T mutations at TE sites was not statistically different from that observed in other regions (P = 0.16). Consequently, a RIP-like mechanism seems unlikely for the L27 hypermutability, and it seems more likely that mutations at loci associated with DNA repair functions are the cause of its high mutation rate and its SNM spectrum (Figure S14b). Future functional studies on the candidate genes that we identified (Table S5) may help to identify genes involved in DNA repair, and related to hypermutability.

References

Böndel KB, Kraemer SA, Samuels T, McClean D, Lachapelle J, Ness RW, Colegrave N, Keightley PD. 2019. Inferring the distribution of fitness effects of spontaneous mutations in Chlamydomonas reinhardtii. PloS Biol. 17 (6): e3000192.

Clutterbuck AJ. 2011. Genomic evidence of repeat-induced point mutation (RIP) in filamentous ascomycetes. Fungal Genet. Biol. 48 (3): 306-326.

Drake JW, Charlesworth B, Charlesworth D, Crow JF. 1998. Rates of spontaneous mutation. Genetics 148 (4): 1667-1686.

Galagan JE, Selker EU. 2004. RIP: the evolutionary cost of genome defense. Trends Genet. 20 (9): 417-423.

Keightley PD, Lynch M. 2003. Toward a realistic model of mutations affecting fitness. Evolution 57 (3): 683-685.

Kraemer SA, Böndel KB, Ness RW, Keightley PD, Colegrave N. 2017. Fitness change in relation to mutation number in spontaneous mutation accumulation lines of Chlamydomonas reinhardtii. Evolution 71 (12): 2918-2929.

Mehta HH, Prater AG, Beabout K, Elworth RAL, Karavis M, Gibbons HS, Shamoo Y. 2019. The essential role of hypermutation in rapid adaptation to antibiotic stress. Antimicrob. Agents Chemother. 63 (7): e00744-19.

Ness RW, Morgan AD, Vasanthakrishnan RB, Colegrave N, Keightley PD. 2015. Extensive de novo mutation rate variation between individuals and across the genome of Chlamydomonas reinhardtii. Genome Res. 25: 1739-1749.

Pal C, Maciá MD, Oliver A, Schachar I, Buckling A. 2007. Coevolution with viruses drives the evolution of bacterial mutation rates. Nature 450 (7172): 1079-1081.

Veschetti L, Sandri A, Johansen HK, Lleò MM, Malerba G. 2020. Hypermutation as an evolutionary mechanism for Achromobacter xylosoxidans in cystic fibrosis lung infection. Pathogens 9 (2): 72.
